# Protein-coding circular RNA enhances antiviral immunity via JAK/STAT pathway in *Drosophila*

**DOI:** 10.1128/mbio.01469-24

**Published:** 2024-08-19

**Authors:** Dongyang Guo, Wen Xu, Ting Cui, Qiqi Rong, Qingfa Wu

**Affiliations:** 1 Department of Pharmacy, The First Affiliated Hospital of USTC, Division of Life Sciences and Medicine, University of Science and Technology of China, Hefei, Anhui, China; 2 Key Laboratory of Anhui Province for Emerging and Reemerging Infectious Diseases, University of Science and Technology of China, Hefei, China; 3 Division of Molecular Medicine, CAS Key Laboratory of Innate Immunity and Chronic Disease, University of Science and Technology of China, Hefei, Anhui, China; University of Würzburg, Würzburg, Germany; French National Centre for Scientific Research, Strasbourg, France

**Keywords:** antiviral immunity, RNAi immunity, protein-coding circRNA, JAK-STAT pathway, *Drosophila *C virus, *Drosophila melanogaster*

## Abstract

RNA interference (RNAi) drives powerful antiviral immunity in plants and animals so that many viruses must express viral suppressor of RNAi (VSR) to establish virulent infection. However, little is known about the immune responses conferring resistance against viruses that have evolved the counter-defensive strategy to suppress antiviral RNAi. In this study, we discover that *Drosophila* cells infected with *Drosophila* C virus (DCV), a natural viral pathogen of *Drosophila* known to harbor a potent VSR, exhibit heightened expression of circular RNA circZfh1. circZfh1 confers virus resistance in the presence of viral suppression of antiviral RNAi. Furthermore, we validate that circZfh1 encodes a 274-amino acid protein, CRAV, essential for its antiviral activity. Notably, CRAV differs from its parental Zfh1 gene in a different reading frame, with the C-terminal 69 amino acids unique to CRAV. Our analysis also reveals the presence of CRAV in species within the melanogaster subgroup, with the C-terminal unique fragment undergoing accelerated evolution. Expression of CRAV upregulates the expression of the cytokine Upd3, which binds to its receptor, stimulating the JAK-STAT pathway and enhancing the immune response to DCV infection. Notably, CRISPR/Cas9 knockout of circZfh1 significantly enhances DCV replication *in vitro* and *in vivo*, with circZfh1-knockout adult flies displaying heightened disease susceptibility to DCV. In summary, our findings unveil a *Drosophila* protein-coding circular RNA that activates an innate immune signaling pathway crucial for virus resistance following the suppression of antiviral RNAi by viruses, thereby elucidating a novel counter-defensive strategy.

**IMPORTANCE:**

Eukaryotic hosts possess a complex, multilayered immune system that guards against pathogen invasion. In fruit flies, RNA interference (RNAi) drives robust antiviral immunity, prompting many viruses to express viral suppressors of RNAi (VSRs) to establish virulent infections. However, little is known about immune responses that confer resistance against viruses with potent VSRs. In this study, we discovered that *Drosophila* cells infected with *Drosophila* C virus (DCV), a natural viral pathogen possessing a potent VSR, upregulated the expression of circular RNA circZfh1. circZfh1 exhibits DCV-specific antiviral activity, encoding a 274-amino acid protein, CRAV, crucial for its antiviral effects. As a different reading frame from its parental Zfh1 gene, the C-terminal 69 amino acids are unique to CRAV, undergoing faster evolution. CRAV activates the JAK-STAT pathway, enhancing the immune response to DCV infection. Therefore, our work uncovers a new strategy for suppressing viral counter-defense through protein-coding circular RNA in fruit flies.

## INTRODUCTION

Antiviral RNA interference (RNAi) is an RNA-based immune mechanism with the specificity programmed by virus-derived small interfering RNAs (siRNAs). Antiviral RNAi plays a central role in the control of virus infections in *Drosophila melanogaster* and other invertebrates as well as in plants and fungi (1–4). Recent studies began to document an antiviral role of the conserved RNAi pathway against distinct RNA viruses in mammals (5–7). In support of a major antiviral function of RNAi, many viruses encode essential virulence proteins that promote infection by inhibiting antiviral RNAi (2, 5–9). Known as viral suppressors of RNAi (VSRs), this group of viral proteins is very diverse in sequence and structure and targets distinct steps of the RNAi pathway (2, 8, 9). Interestingly, all confirmed mammalian VSRs from four families of RNA viruses are dsRNA-binding proteins and suppress Dicer processing of virus-specific dsRNA into siRNAs (5–7, 10, 11), similar to the B2 and 1A proteins encoded by insect viruses Flock house virus (FHV) and *Drosophila* C virus (DCV), respectively (1, 4, 12).

Less is known about the immune responses that confer antiviral protection against viruses that have evolved the counter-defensive strategy to suppress antiviral RNAi. In plants, programmed cell death (hypersensitive response) is induced to limit virus spread by VSRs such as 2b of Tomato aspermy cucumovirus and NSs of Tomato spotted wilt virus (13, 14). In adult mice, either the adaptive immunity mediated by B and T lymphocytes or the STAT1/STAT2-dependent innate immunity becomes essential for the clearance of Nodamura virus (NoV), but not NoV mutants defective in RNAi suppression by its VSR B2 protein (11, 15).

*D. melanogaster* serves as a valuable model for investigating innate immunity mechanisms (16), especially because it shares essential innate immune components with humans, including highly conserved JAK/STAT, Toll, Imd, and STING signaling pathways (17). Although the antiviral RNAi pathway targets both DNA and RNA viruses, these inducible innate immune pathways appear to initiate virus-specific antiviral responses (17–23). For instance, the Toll pathway in flies mediates resistance to DCV, *Drosophila* X virus (DXV), Cricket paralysis virus (CrPV), FHV, and Nora virus (18, 22). Mutations in the Imd pathway reduce resistance to Sindbis virus (SINV) and CrPV (20, 21). The STING pathway defends against DCV and CrPV (24), whereas the JAK-STAT pathway plays a crucial role in the antiviral response against DCV, CrPV, and invertebrate iridescent virus 6 (IIV-6) (17, 19, 25). We have recently identified a *Drosophila* long noncoding RNA that activates a noncanonical antimicrobial defense pathway by directly interacting with DCV-1A, the VSR protein involved in DCV counter-defense (26). We thus hypothesize that additional antiviral responses are induced in *Drosophila* in response to viral suppression of the primary antiviral mechanism directed by RNAi.

Circular RNAs (circRNAs) are noncoding RNAs that are widely expressed and play diverse roles in various biological processes in eukaryotes (27). In mammals, circRNAs have been found to participate in immune responses, such as inhibiting the double-stranded RNA-dependent protein kinase (PKR) and regulating the antiviral activity of NF90/NF110 (28) as well as blocking the enzymatic activity of the DNA sensor cGAS (29). However, only a few circRNAs have been functionally characterized in *Drosophila*. The splicing factor MBL binds within its primary RNA, promoting circMbl biogenesis while concurrently decreasing linear splicing, suggesting that circMbl functions in gene regulation through competition with linear splicing (30). CircBoule, derived from the evolutionarily conserved reproductive gene Boule across species from *Drosophila* to humans, serves as a critical modulator of male reproductive function in animals (31). Recent studies have discovered that some circRNAs encode proteins and undergo translation in a cap-independent manner (32–34). CircSfl is consistently upregulated in the brain and muscle tissues of long-lived fruit flies, and its translation into a protein significantly extends lifespan (35). Notably, brain-enriched circRNA Edis (circ_Ect4) encodes a protein that suppresses the processing and activation of the immune transcription factor Relish, thus regulating innate immunity and neurodevelopment in *Drosophila* (36). However, the potential roles of circRNAs in the immune responses of fruit flies remain largely unexplored.

In this study, we aimed to explore the role of circRNAs in the immune response to DCV, a natural viral pathogen of *Drosophila* from the *Dicistroviridae* family known to harbor a potent VSR (DCV-1A). We discovered an upregulation of circZfh1 in *Drosophila* cells infected with DCV, and we found that circZfh1 exhibits an antiviral role against DCV infection. Moreover, we discovered that circZfh1 encodes a 35kD protein, namely CRAV, and the antiviral activity of circZfh1 is dependent on CRAV. Importantly, our results demonstrated that the expression of CRAV increases the expression of Upd3 and triggers the JAK/STAT pathway to enhance antiviral function. These findings provide insights into the circRNA-induced immune response that protects against *Drosophila* viral pathogens capable of potent suppression of the primary antiviral RNAi response.

## RESULTS

### DCV infection activates circZfh1-dependent virus resistance

**Fig 1:**
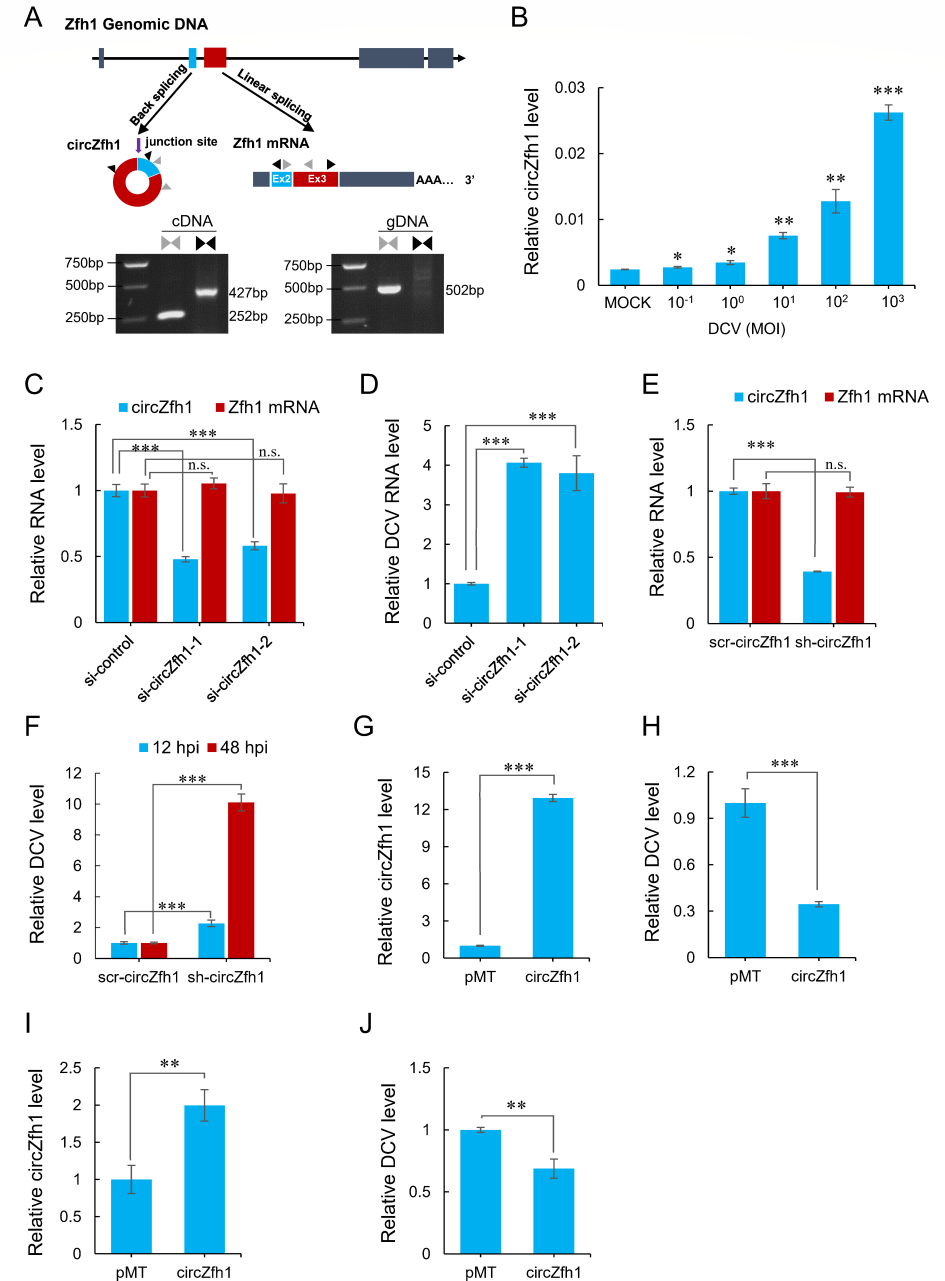
DCV infection induced circZfh1. (**A**) Upper panel: genomic structure of Zfh1 with illustration of putative alternative RNA splicing forms and validation strategy for circular exon 2 and 3 (circZfh1). Convergent (gray) and divergent (black) primer sets were designed to amplify linear or back-splicing products. Lower panel: The convergent and divergent PCR products amplified on gDNA and cDNA from S2 cells are shown. (**B**) RT-qPCR analysis of circZfh1 expression levels, normalized to *rp49*, in S2 cells infected with different concentrations of DCV at 48 hpi. (**C**) RT-qPCR analysis of circZfh1 and Zfh1 mRNA expression levels, normalized to rp49, in S2 cells transfected with control siRNA or two siRNAs targeting circZfh1 for 48 h. (**D**) S2 cells transfected with control siRNA, si-circZfh1-1, or si-circZfh1-2 for 48 h were infected with DCV, followed by RT-qPCR analysis of DCV RNA levels at 48 hpi, normalized to *rp49*. (**E**) RT-qPCR analysis of circZfh1 and Zfh1 mRNA expression levels, normalized to rp49, in scr-circZfh1 or sh-circZfh1 cells. (**F**) RT-qPCR analysis of relative DCV RNA levels, normalized to *rp49*, in scr-circZfh1, or sh-circZfh1 cells infected with DCV at 12 and 48 hpi. (**G**) RT-qPCR analysis of circZfh1 expression in S2 cells transfected with the exogenous expression plasmids of circZfh1 or pMT empty vector. (**H**) S2 cells transfected with pMT or pMT-circZfh1 plasmids were infected with DCV, followed by RT-qPCR analysis of DCV RNA levels, normalized to *rp49*, at 48 hpi. (**I and J**) The sh-circZfh1 cells transfected with pMT (control) or pMT-circZfh1 plasmids were infected with DCV, prior to RT-qPCR analysis of circZfh1 (**I**) and DCV RNA levels (**J**), normalized to rp49, at 48 hpi. The mean ± SD of three independent experiments is shown (**B–J**); statistical analysis was performed for panels **B–J**; *P < 0.05; **P < 0.01; ***P < 0.001; n.s., not significant (Student’s *t-*test). See also Fig. S1.

We conducted an analysis of RNA-seq libraries from *Drosophila* S2 cells to characterize circRNAs associated with virus infection. Our findings revealed that some circRNA levels were downregulated or upregulated by DCV infection (Fig. S1A). Among these regulated circRNAs, we identified a circular RNA that was significantly upregulated in DCV-infected S2 cells, designated as circZfh1 (Fig. S1B). circZfh1 is 875 nucleotides long and belongs to the exon-type circRNA, formed by the second and third exons of the gene encoding the transcription factor Zfh1 (Fig. 1A). Production of circZfh1 was confirmed by RT-PCR utilizing a divergent primer pair followed by Sanger sequencing (Fig. 1A; Fig. S1C). Consistent with the analysis of RNA-seq libraries, we found that the copy count of circZfh1 per cell was approximately 14 in S2 cells and increased to 50 in DCV-infected cells (Fig. S1D). Further investigation revealed a positive correlation between the expression level of circZfh1 in cells and the concentration of DCV inoculum (Fig. 1B).

To explore the antiviral role of circZfh1 in S2 cells, we designed and synthesized two distinct siRNAs to target the junction region of circZfh1 for specific knockdown of circZfh1 without altering the expression of its parental Zfh1 mRNA and ZFH1 protein levels (Fig. 1C; Fig. S1E). We found that circZfh1 knockdown by si-circZfh1 resulted in a significant increase in DCV RNA levels (Fig. 1D) and viral titers (Fig. S1F). Additionally, we established stable S2 cell lines expressing a short hairpin RNA (shRNA) to target the same junction sequence of circZfh1 (sh-circZfh1) and control cell lines expressing scrambled shRNA (scr-circZfh1). Notably, the expression of circZfh1 was significantly reduced in sh-circZfh1 cells, whereas no alterations were observed in both Zfh1 mRNA and ZFH1 protein levels (Fig. 1E; Fig. S1G). Moreover, we evaluated the expression levels of predicted off-target genes in sh-circZfh1 cells and found no evidence of off-target effects (Fig. S1H). Following DCV infection, sh-circZfh1 cells exhibited higher viral RNA levels or viral titers compared with scr-circZfh1 cells at both 12 and 48 h post-infection (hpi) (Fig. 1F; Fig. S1I).

We also constructed a circZfh1 expression plasmid, pMT-circZfh1, which efficiently produced circZfh1 in S2 cells (Fig. S1J; Fig. 1G). The exogenous expression of circZfh1 by transfecting pMT-circZfh1 into cells and subsequently challenging them with DCV virus resulted in a significant reduction in DCV replication (Fig. 1H). Furthermore, introducing pMT-circZfh1 into sh-circZfh1 cells restored circZfh1 expression and effectively inhibited DCV replication (Fig. 1I and J). These findings suggest that upregulation of circZfh1 in response to DCV infection has the ability to restrict DCV replication in *Drosophila* cells.

### circZfh1 enhances virus resistance in the presence of antiviral RNAi suppression

**Fig 2:**
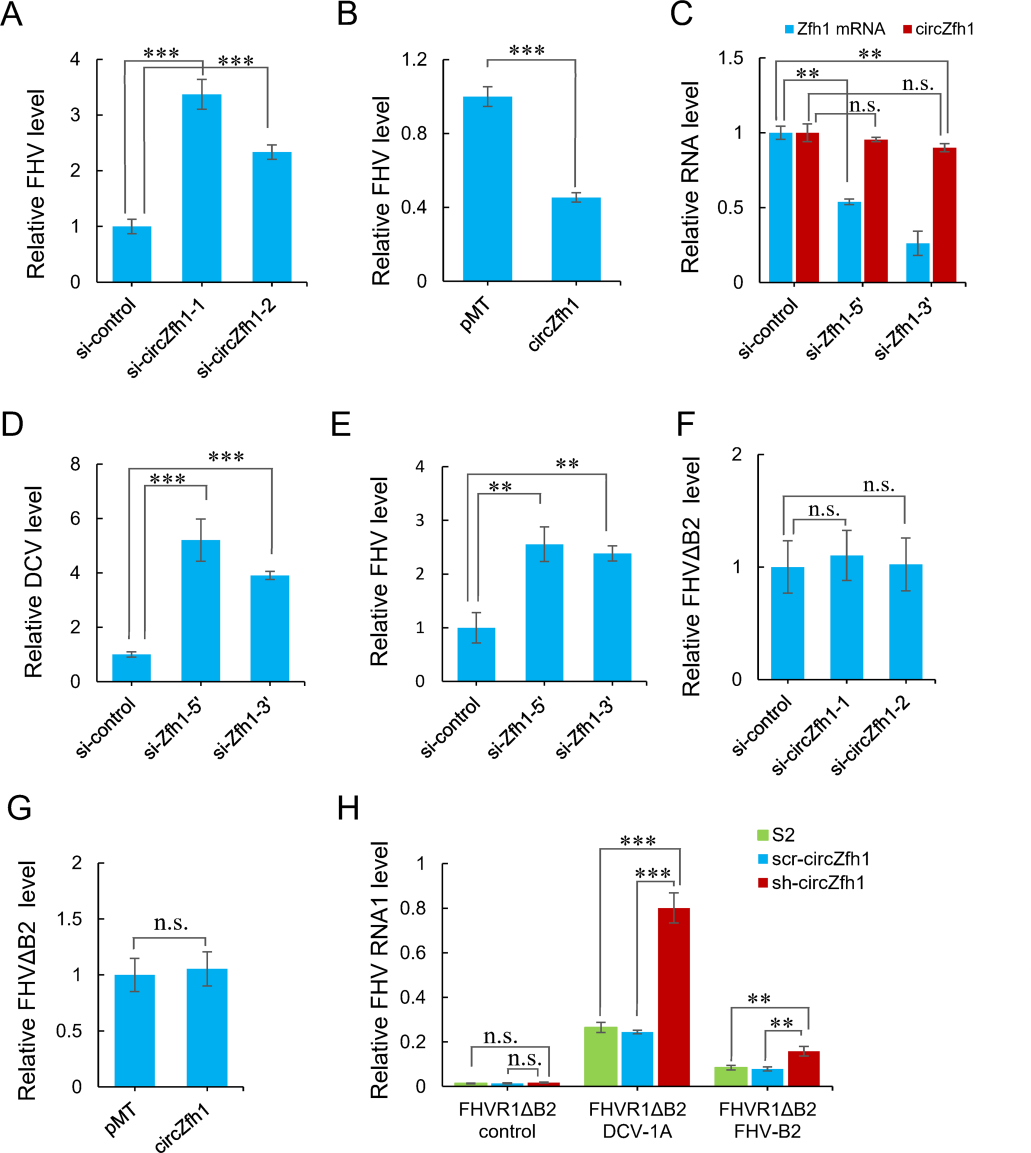
circZfh1 enhances virus resistance in the presence of antiviral RNAi suppression. (**A**) S2 cells transfected with control siRNA, si-circZfh1-1, or si-circZfh1-2 for 48 h were infected with FHV (MOI = 1), followed by RT-qPCR analysis of FHV RNA levels at 48 hpi, normalized to *rp49*. (**B**) S2 cells transfected with pMT or pMT-circZfh1 plasmids were infected with FHV, followed by RT-qPCR analysis of FHV RNA levels, normalized to *rp49*, at 48 hpi. (**C**) RT-qPCR analysis of circZfh1 and Zfh1 mRNA expression levels, normalized to rp49, in S2 cells transfected with control siRNA or two siRNAs targeting Zfh1 mRNA for 48 h. (**D**) S2 cells transfected with control siRNA, si-Zfh1-5’, or si-Zfh1-3’ for 48 h were infected with DCV, followed by RT-qPCR analysis of DCV RNA levels at 48 hpi, normalized to *rp49*. (**E**) S2 cells transfected with control siRNA, si-Zfh1-5’, or si-Zfh1-3’ for 48 h were infected with FHV, followed by RT-qPCR analysis of FHV RNA levels at 48 hpi, normalized to *rp49*. (**F**) S2 cells transfected with control siRNA, si-circZfh1-1, or si-circZfh1-2 for 48 h were infected with FHVΔB2, followed by RT-qPCR analysis of FHVΔB2 RNA levels at 24 hpi, normalized to *rp49*. (**G**) S2 cells transfected with pMT or pMT-circZfh1 plasmids were infected with FHVΔB2, followed by RT-qPCR analysis of FHVΔB2 RNA levels, normalized to *rp49*, at 24 hpi. (**H**) FHV RNA1 levels were measured in S2 cells, scr-circZfh1 cells, and sh-circZfh1 cells co-transfected with pMT-FR1ΔB2 and pMT-DCV-1A, pMT-FHV B2 or control plasmids at 48 h post-transfection and shown as relative to the wild-type controls. The mean ± SD of three independent experiments is shown (**A–H**); statistical analysis was performed for panels **A–H**; **P < 0.01; ***P < 0.001; n.s., not significant (Student’s *t-*test). See also Fig. S2.

We observed no upregulation of circZfh1 in S2 cells in response to the infection with FHV (Fig. S2A), unlike DCV infection (Fig. S1D). Nevertheless, FHV replication was significantly enhanced following circZfh1 knockdown using circZfh1-specific siRNA in S2 cells (Fig. 2A) or in the stable sh-circZfh1 cells (Fig. S2B). Additionally, ectopic expression of circZfh1 inhibited FHV replication (Fig. 2B). Considering that the parental gene Zfh1 negatively modulates the Imd pathway, leading to an increase in the expression of marker genes, such as *Attacin D* (*AttD*) and *Diptericin* (*DptB*), upon Zfh1 knockdown in *Drosophila* S2 cells (37), we designed siRNAs targeting the 5' or 3' UTRs of Zfh1 mRNA to specifically reduce Zfh1 mRNA levels without significantly affecting circZfh1 expression (Fig. 2C). Importantly, we observed an increase in both DCV and FHV levels in S2 cells upon Zfh1 mRNA knockdown (Fig. 2D and E), accompanied by higher expression of *AttD* and *DptB* marker genes (Fig. S2C). In contrast, we noted no significant changes in the expression of these marker genes in circZfh1 knockdown cells infected with DCV and FHV (Fig. S2D), suggesting that circZfh1 restricts virus replication independently from the Imd pathway.

In stark contrast to wild-type DCV or FHV, the stable and transient knockdown of circZfh1 as well as the ectopic expression of circZfh1 all had no significant effect on the infection with FHVΔB2, a FHV mutant not expressing its VSR-B2 protein (1) (Fig. 2F and G; Fig. S2E). Moreover, the self-replicating FHV RNA1 mutant not expressing its B2 protein (FR1ΔB2) also replicated to similar levels in wild-type S2 cells, scr-circZfh1 cells, and sh-circZfh1 cells (Fig. 2H). Consistent with previous studies (1), the FR1ΔB2 replicon replicated to higher levels in these S2 cells after RNAi suppression by B2 of FHV ectopically expressed from a co-transfected plasmid (Fig. 2H). The rescue of FR1ΔB2 in these S2 cells was much more efficient by ectopic expression of DCV-1A than FHV-B2 (Fig. 2H). Due to the stable knockdown of circZfh1 significantly enhancing the replication of FR1ΔB2 in the presence of RNAi suppression by either FHV-B2 or DCV-1A (Fig. 2H), these findings suggest that circZfh1 confers virus resistance in the presence, but not in the absence, of viral suppression of antiviral RNAi in S2 cells, thereby revealing a new counter counter-defensive strategy.

### A 35 kDa protein translated from both endogenous and ectopically expressed circZfh1

**Fig 3:**
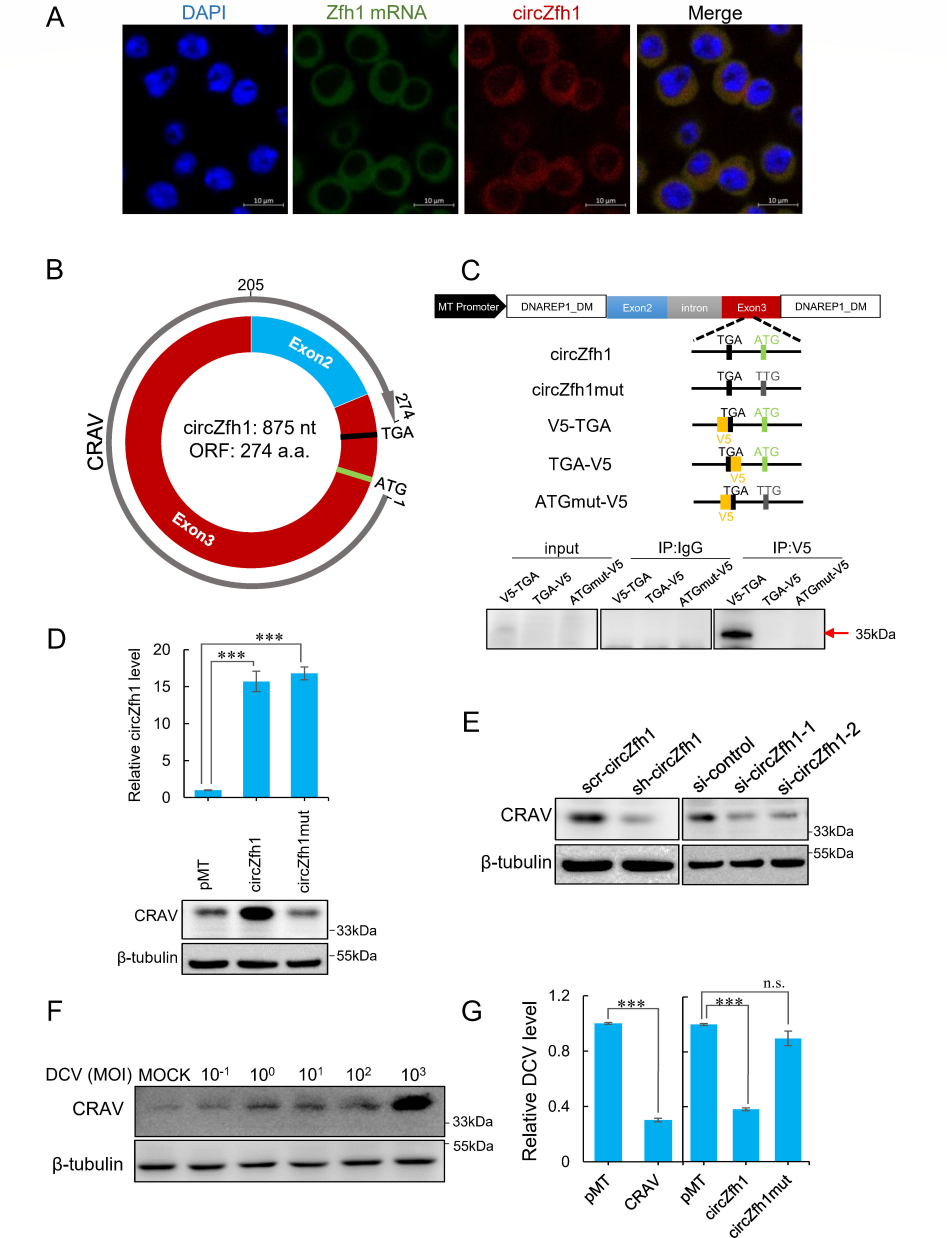
circZfh1-dependent virus resistance is mediated by its coding protein CRAV. (**A**) Subcellular localization of circZfh1 and Zfh1 mRNA in S2 cells is revealed by FISH. Red fluorescence indicates circZfh1, green fluorescence indicates Zfh1 mRNA, and blue fluorescence indicates nuclear location stained by DAPI. Scale bar = 10 µm. (**B**) Illustration of the protein encoded by circZfh1 with annotated ORF positional information. (**C**) Upper panel: illustrations of exogenous expression vectors of circZfh1 and mutants. pMT-circZfh1: Illustration showing the original circZfh1 exogenous expression vector. The predicted start codon (ATG) and stop codon (TGA) are shown. pMT-circZfh1mut: the start codon ATG mutated to TTG in pMT-circZfh1. V5-TGA: The V5 tag was added to the front of TGA. TGA-V5: The V5 tag added behind the TGA in pMT-circZfh1. ATGmut-V5: the V5 tag was added to the front of TGA in pMT-circZfh1mut. Lower panel: S2 cells transfected with V5-TGA, TGA-V5, and ATGmut-V5 subjected to IP using anti-V5 antibody and Western blot for IP pellets using anti-V5 antibody. (**D**) S2 cells transfected with pMT-circZfh1, pMT-circZfh1mut, or pMT empty plasmid for 48 h, followed by RT-qPCR for relative circZfh1 levels normalized to *rp49* (top) and Western blot for CRAV protein quantification using anti-CRAV antibody(bottom). (**E**) Western blot for CRAV in scr-circZfh1 or sh-circZfh1 cells and S2 cells transfected with si-control, si-circZfh1-1, or si-circZfh1-2. (**F**) S2 cells infected with DCV at different concentrations were subjected to Western blotting for CRAV at 48 hpi. (**G**) S2 cells transfected with pMT, pMT-CRAV, pMT-circZfh1, or pMT-circZfh1mut infected with DCV (MOI = 1), followed by RT-qPCR analysis of DCV RNA levels normalized to *rp49* at 48 hpi. The mean ± SD of three independent experiments is shown (**D and G**); statistical analysis was performed for panels D and G; ***P < 0.001; n.s., not significant (Student’s *t-*test). The representatives of triplicate experiments are shown (**C–F**). See also Fig. S3.

We used FISH to visualize circZfh1 and its parental mRNA in S2 cells. The results showed that both RNAs were mostly present in the cytoplasm of S2 cells (Fig. 3A), suggesting that circZfh1 could have access to ribosomes for protein translation. Moreover, we observed a substantial reduction in the FISH signal intensity of circZfh1 in siRNA-treated cells and sh-circZfh1 cells compared with the control cells (Fig. S3A and B).

We discovered a circZfh1-encoded open reading frame (ORF) that spans the circZfh1 junction site, consisting of 274 amino acids (Fig. 3B). To verify the ability to translate the ORF, we constructed the V5-TGA, TGA-V5, and ATGmut-V5 expression vectors based on the pMT-circZfh1 vector (Fig. 3C). Indeed, we detected a protein band of approximately 35 kDa in S2 cells transfected with one of the circZfh1 expression plasmids (V5-TGA) with the V5 tag inserted before the predicted stop codon (Fig. 3C). As expected, no protein band was detected in TGA-V5 or ATGmut-V5-transfected cells (Fig. 3C). Thus, we conclude that the ORF encoded by circZfh1 is translated into a protein, designated as the circular RNA-encoded antiviral protein (CRAV).

Polyclonal antibodies were generated to target either the entire CRAV protein (Ab1) or specific peptides (Ab4, Ab217) located in the N- and C-terminal regions, respectively (Fig. S3C). CRAV was detectable by all of the three antibodies in S2 cells transfected with the V5-tagged circZfh1 expression plasmid (V5-TGA) described above (Fig. S3D). We further transfected S2 cells with plasmids pMT, pMT-circZfh1, or pMT-circZfh1mut created by mutating the start codon ATG to TTG (Fig. 3C). RT-qPCR showed a significant increase in circZfh1 RNA levels in cells transfected with either pMT-circZfh1 or pMT-circZfh1mut compared with the control cells (Fig. 3D). The Ab1 antibody was able to detect a band of the corresponding size in control cells, indicating that endogenous circZfh1 can encode CRAV (Fig. 3D). CRAV accumulated at similar levels in control group and cells transfected with pMT-circZfh1mut, whereas a significantly increased CRAV accumulation was observed in S2 cells transfected with pMT-circZfh1 (Fig. 3D; Fig. S3E). Moreover, CRAV accumulated to lower levels following transient or stable circZfh1 knockdown (Fig. 3E; Fig. S3F). All the above results confirmed translation of the 35 kDa CRAV protein in S2 cells encoding by the circular RNA circZfh1.

### circZfh1-dependent virus resistance is mediated by its encoded protein CRAV

We investigated whether CRAV contributes to the antiviral activity of circZfh1. We found that CRAV levels were upregulated by DCV infection and positively correlated with the concentration of DCV inoculum (Fig. 3F; Fig. S3G), consistent with the upregulation of circZfh1 RNA levels (Fig. 1B). Compared with control cells transfected with pMT, cells transfected with pMT-CRAV or pMT-circZfh1 exhibited significantly lower levels of DCV replication, whereas cells transfected with pMT-circZfh1mut showed no such reduction (Fig. 3G), despite comparable levels of wild-type and mutant circular RNA in cells transfected with pMT-circZfh1mut or pMT-circZfh1 (Fig. 3D). These findings indicate that the antiviral activity of circZfh1 is mediated by its encoded protein CRAV. Consistent with the circZfh1 results, ectopic expression of CRAV markedly inhibited FHV replication but had no significant effect on FHVΔB2 infection, suggesting that CRAV confers virus resistance only in the presence, but not in the absence, of viral suppression of antiviral RNAi in S2 cells (Fig. S3H and I).

### The novel C-terminal region of CRAV undergoes faster evolution

**Fig 4:**
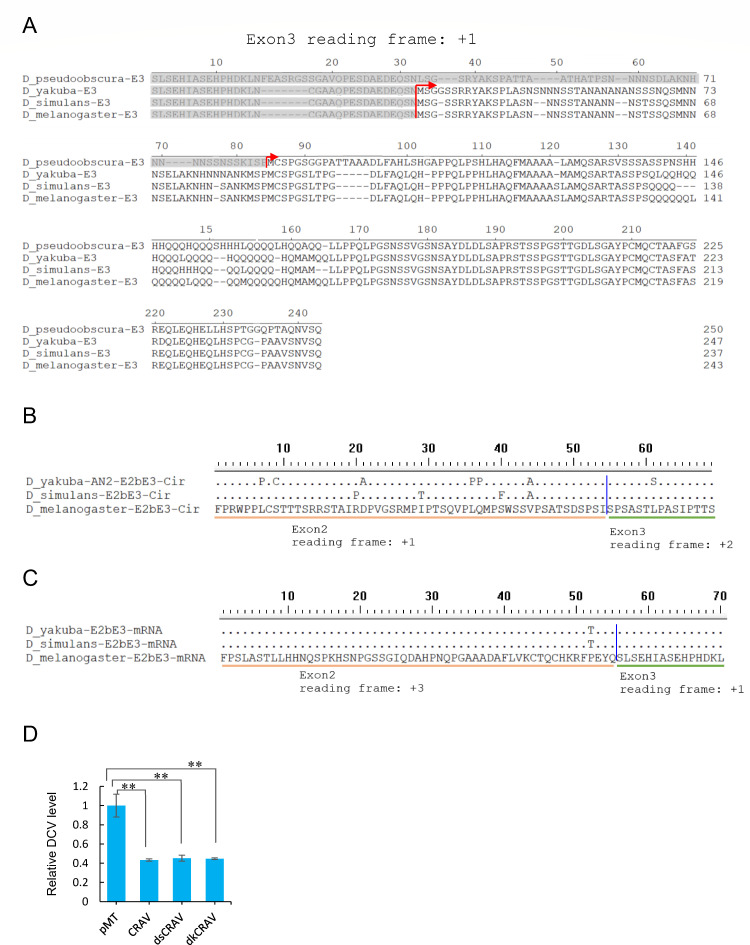
The novel C-terminal region of CRAV undergoes faster evolution (**A**) Sequence alignment of exon3-encoded protein fragments of ZFH1 in *D. simulans*, *D*.*melanogaster*, *D. yakuba*, and *D. pseudoobscura*, with the start codon of CRAV indicated by the red line. (**B and C**) Sequence alignment of the unique C-terminal ends of CRAV (**B**) or corresponding ZFH1 protein fragments (**C**) in *D. simulans*, *D. melanogaster*, *D. yakuba,* and *D. pseudoobscura*, with exon2 and exon3 reading frames depicted below. (**D**) S2 cells transfected with pMT, pMT-CRAV, pMT-dsCRAV, or pMT-dkCRAV plasmids were infected with DCV, followed by RT-qPCR analysis of DCV levels, normalized to *rp49*, at 48 hpi. The mean ± SD of three independent experiments is shown (**D**); statistical analysis was performed for panel **D**; **P < 0.01 (Student’s *t*-test). See also Fig. S4.

We examined the potential formation of the protein-encoding circZfh1 through back splicing of exon 2 and exon 3 in *Drosophila simulans*, *D. melanogaster*, and *Drosophila yakuba* within the melanogaster subgroup, as well as in *Drosophila pseudoobscura* within the closest obscura group. As expected, RT-PCR and Sanger sequencing confirmed the exclusive presence of circZfh1 in these species (Fig. S4A). Additionally, we observed the conservation of the initial ATG codon for CRAV translation in *D. simulans*, *D. melanogaster*, and *D. yakuba*, whereas *D. pseudoobscura* exhibited a leucine-encoding mutation (Fig. 4A). These results suggested that circZfh1 encodes a protein of similar size in *D. simulans*, *D. melanogaster*, and *D. yakuba*.

The reading frame of exon 3 for the Zfh1 protein remains consistent at +1 in all species, with protein similarity distances among them reflecting the species tree (Fig. S4B and C). In comparison to its parental Zfh1 gene, the CRAV-coding frame begins similarly in exon 3 but undergoes a shift to a different reading frame in exon 2 and the 5'-end of exon 3 (Fig. 4B and C). Consequently, the N-terminal 205 amino acids of CRAV mirror those of Zfh1, with the addition of a novel C-terminal region spanning 69 amino acids. Subsequently, we investigated the conservation of the unique C-terminal end of CRAV and identified 7 and 4 mutations in *D. yakuba* and *D. simulans*, respectively, compared with *D. melanogaster* (Fig. 4B). Further analysis of peptides translated from the corresponding part of Zfh1 mRNA revealed fewer mutations, with only one mutation in *D. yakuba* and *D. simulans* each, compared with *D. melanogaster* (Fig. 4C). These results suggest that CRAV evolves at a faster rate than Zfh1 proteins. These results suggest that the novel C-terminal region of CRAV undergoes faster evolution compared with the Zfh1 protein.

We further investigated whether CRAV from *D. simulans* (dsCRAV) or *D. yakuba* (dkCRAV) also possesses antiviral activity. The results indicated that ectopic expression of dsCRAV and dkCRAV similarly inhibited DCV replication (Fig. 4D), suggesting conservation of the antiviral function of CRAV across all species within the melanogaster subgroup.

### Activation of the JAK-STAT pathway by CRAV boosts the immune response

**Fig 5:**
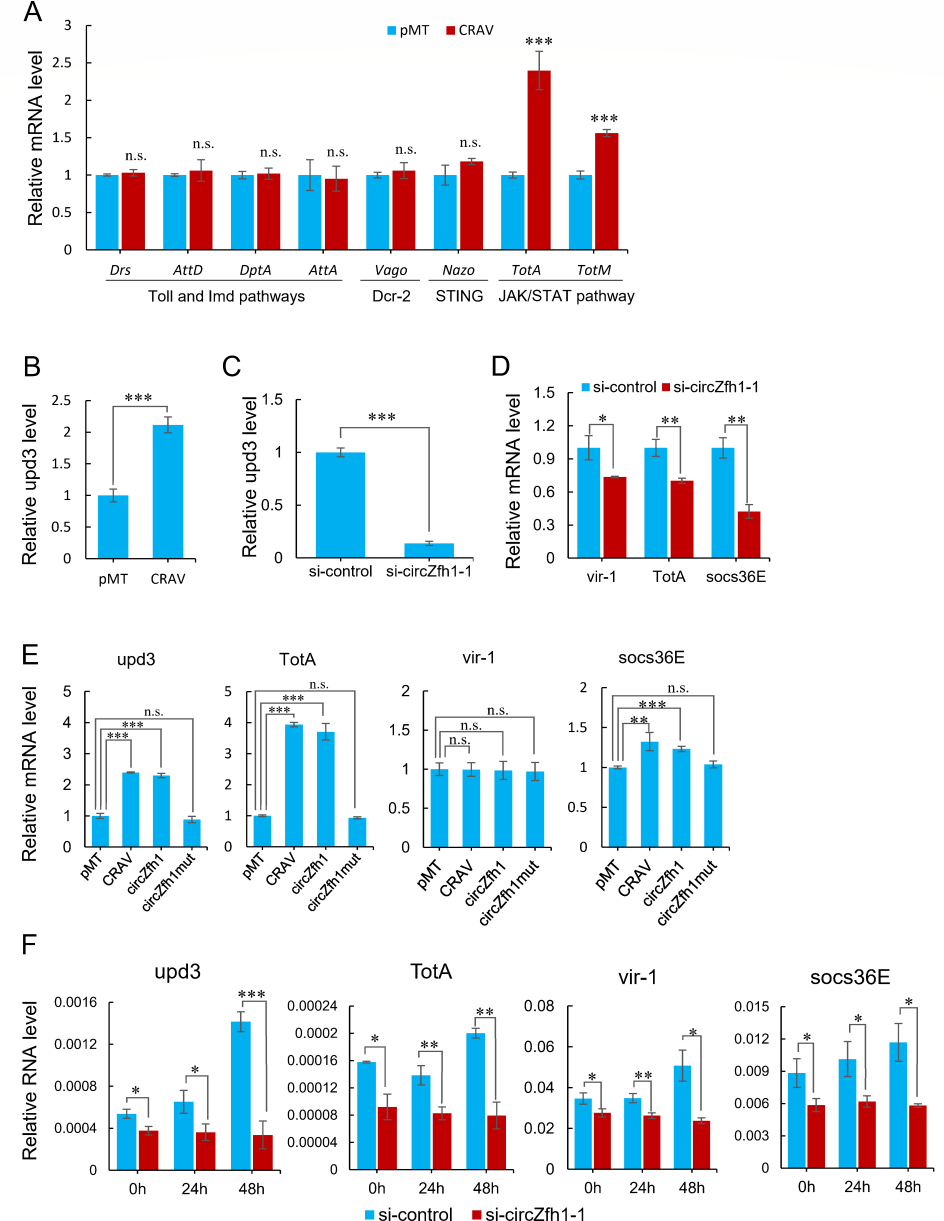
Activation of the JAK-STAT pathway by CRAV boosts the immune response. (**A**) S2 cells were transfected with pMT vector and pMT-CRAV vector infected with DCV (MOI = 1) followed by RT-qPCR analysis of the indicated gene levels normalized to *rp49* at 48 hpi. (**B**) RT-qPCR analysis of relative mRNA levels of *upd3* in S2 cells transfected with pMT or pMT-CRAV. (**C**) S2 cells were transfected with si-circZfh1-1 and si-control, followed by RT-qPCR to determine the relative mRNA levels of *upd3*. (**D**) S2 cells were transfected with si-circZfh1-1 and si-control, followed by RT-qPCR to determine the relative mRNA levels of *vir-1*, *TotA,* and *socs36E*. (**E**) S2 cells were transfected with plasmids of pMT, pMT-CRAV, pMT-circZfh1, or pMT-circZfh1mut, respectively, followed by RT-qPCR to determine the relative mRNA levels of *upd3*, *TotA*, *vir-1,* and *socs36E*. (**F**) RT-qPCR analysis of relative mRNA levels of *upd3*, *TotA*, *vir-1,* and *socs36E* in S2 cells pretransfected with si-control or si-circZfh1-1 and infected with DCV for 24 and 48 h. The mean ± SD of three independent experiments is shown (**A–F**); statistical analysis was performed for panels **A–F**; *P < 0.05; **P < 0.01; ***P < 0.001; n.s., not significant (Student’s *t*-test). See also Fig. S5.

To investigate the antiviral mechanism of CRAV, we transfected pMT-CRAV into S2 cells followed by DCV infection for 48 h, then detected the expression levels of makers genes associated with the known antiviral pathways in response to DCV infection. The results showed that the antiviral response mediated by CRAV was not associated with Dcr-2/Vago, STING/Nazo, Toll, and Imd pathways (Fig. 5A). However, the expression of *TotA* and *TotM*, target genes of the JAK/STAT pathway, was significantly up-regulated upon ectopic expression of CRAV (Fig. 5A).

The mammalian JAK/STAT pathway is part of an important signaling cascade that regulates both the antiviral immune response and inflammatory cytokine signaling (38). The major components of the conserved JAK/STAT pathway in *Drosophila* include the ligand *upd1/2/3*, the receptor *Domeless*, the JAK kinase *Hopscotch* (*Hop*), and the transcription factor *Stat92E* (39, 40). A previous study showed that the JAK-STAT pathway of *Drosophila* confers antiviral activity against DCV and related viruses in the *Dicistroviridae* (17). We verified the JAK-STAT-dependent inhibition of DCV replication in S2 cells since knockdown of either the transcription factor *Stat92E* or its target gene *TotA* significantly enhanced DCV accumulation (Fig. S5A and B). Indeed, CRAV expression significantly upregulated *upd3* expression in S2 cells, whereas the expression levels of *upd1* and *upd2* showed no significant change (Fig. 5B; Fig. S5C), indicating potential activation of the conserved JAK-STAT pathway by CRAV. In support of the hypothesis, circZfh1 knockdown in S2 cells led to significant downregulation of not only *upd3* expression (Fig. 5C) but also *vir-1*, *TotA,* and *socs36E*, which are representative marker genes of the JAK-STAT pathway(19, 41) (Fig. 5D; Fig. S5D). Moreover, ectopic expression of CRAV or the circular RNA circZfh1, but not circZfh1mut, significantly upregulated the expression levels of *upd3*, *TotA,* and *socs36E in S2 cells* without significantly altering the expression of *vir-1* (Fig. 5E). We further showed that the addition of Upd3-containing culture medium upregulated *TotA* expression in naive cells and inhibited DCV replication (Fig. S5E). These findings suggest that CRAV-induced Upd3 expression activates the JAK-STAT pathway not only in CRAV-expressing cells but also in surrounding cells, thereby conferring resistance to DCV infection.

Furthermore, we found that the expression levels of *upd3*, *vir-1*, *TotA,* and *socs36E* were significantly downregulated in circZfh1 knockdown cells infected with DCV (Fig. 5F). Interestingly, the expression levels of these target genes remained stable at 24 and 48 hpi in the circZfh1 knockdown cells, in contrast to the increased upregulation observed in the control cells (Fig. 5F). Our results revealed that CRAV expression activates the JAK-STAT signaling pathway to enhance antiviral immunity in response to DCV infection.

### circZfh1-dependent resistance inhibited DCV replication in *Drosophila* adults

**Fig 6:**
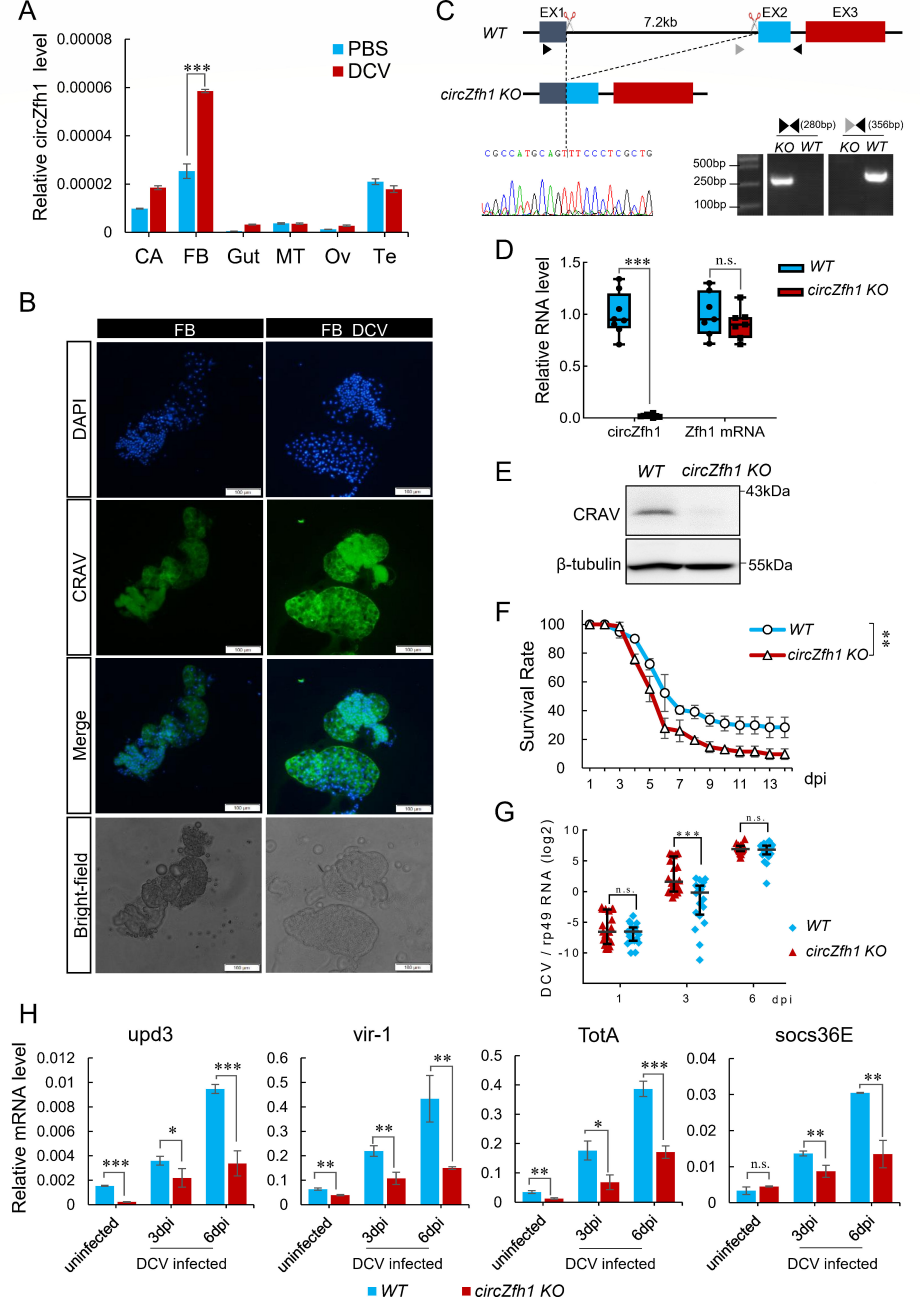
circZfh1 plays a crucial role in the antiviral response of *Drosophila* adults. (**A**) WT flies (*n* = 20) injected with PBS or DCV virions were dissected at 5 dpi, and the relative circZfh1 levels were measured by RT-qPCR in the fat bodies (FB), gut, malpighian tubules (MT), testis (TE), ovary (Ov), and carcass (CA), normalized to *rp49*. (**B**) WT flies were dissected, and the distribution of CRAV in the fat body was detected by immunofluorescence. The green fluorescence signal indicates CRAV, and the blue fluorescence signal indicates the nuclear location stained by DAPI. Scale bar = 100 µm. (**C**) Schematic diagram of circZfh1 knockout (KO) flies constructed using the CRISPR/Cas9 system (top). The circZfh1 KO flies were confirmed by PCR using the indicated primer sets and Sanger sequencing (bottom). (**D**) RT-qPCR analysis of relative circZfh1 and Zfh1 mRNA levels in WT flies and circZfh1 KO flies. (**E**) Western blot for CRAV in *WT* and *circZfh1 KO* flies. (**F and G**) WT and circZfh1 KO flies were injected with PBS or DCV virions. Then, the survival rates were monitored daily (**F**), and the DCV RNA levels, measured in individual flies by RT-qPCR and normalized to *rp49* at the indicated times, are shown as log2 values (**G**). Each dot represents four male flies, *n* = 4´20. (**H**) RT-qPCR analysis of relative mRNA levels of *upd3*, *vir-1*, *TotA*, and *socs36E* in WT flies and circZfh1 KO flies infected with DCV at 3 and 6 dpi. The mean ± SD of three independent experiments is shown (**A, F, H**); Boxplot and dot plot represent the median and interquartile range (**D and G**); statistical analysis was performed for panels **A, D, and F–H**; *P < 0.05; **P < 0.01; ***P < 0.001; n.s., not significant (Student’s *t-*test in panels A, D, G, and H or log-rank test [Kaplan‒Meier method] in panel F).

To investigate the role of circZfh1 *in vivo*, we evaluated its expression levels in various tissues of fruit flies with or without DCV infection. RT-qPCR analysis revealed *in vivo* accumulation of the circular RNA circZfh1, especially in the fat body where circZfh1 expression was further induced significantly by DCV infection (Fig. 6A). Tissue immunofluorescence microscopy revealed an enhanced expression of the CRAV protein in the fat body after DCV infection (Fig. 6B). We utilized the CRISPR/Cas9 technology to delete the intron located between the first and second exon of the *Zfh1* gene, yielding a mutant *Drosophila* strain that selectively inhibited the expression of circZfh1 without significantly altering the parental *Zfh1* mRNA level (Fig. 6C and D). This knockout mutant strain was designated *circZfh1 KO*. We detected CRAV protein in fat body of *WT* and *circZfh1 KO* flies by western blot, and it was only detected in *WT* flies (Fig. 6E).

We compared the survival of wild-type and *circZfh1 KO* flies after DCV infection. We found that the survival of the infected *circZfh1 KO* flies was significantly reduced compared with the infected *WT* flies (Fig. 6F). RT-qPCR analysis revealed that DCV genomic RNA accumulated to significantly higher levels in *circZfh1 KO* flies than WT flies by 3 days post-infection (dpi) (Fig. 6G). Interestingly, the differences in the viral RNA levels became not significant at 6 dpi (Fig. 6G) when more DCV-infected *circZfh1 KO* flies died than DCV-infected *WT* flies (Fig. 6F). RT-qPCR analyses revealed that *upd3*, *vir-1*, *TotA,* and *socs36E* were induced in *WT* flies by DCV infection (Fig. 6H). Notably, the *in vivo* induction of the cytokine and JAK-STAT marker genes was all significantly inhibited at both 3 and 6 dpi in *circZfh1 KO* flies (Fig. 6H) as occurred in S2 cells following circZfh1 knockdown. Together, these results support a model in which the circular RNA circZfh1 confers resistance to DCV infection in adult flies through activation of the JAK-STAT pathway.

## DISCUSSION

Eukaryotic hosts possess a complex multilayered immune system that guards against invasion by pathogens. Primary antiviral RNAi immunity provides robust defense against both DNA and RNA viruses; however, the RNAi pathway is generally suppressed by the virulence factor VSRs (42). To fight virus infection, hosts have evolved diverse mechanisms to sense virus infection and activate the immune response to suppress virus replication, analogous to the “zigzag” model of immunity to nonviral pathogens in plants (43, 44). The JAK-STAT pathway displays a virus-specific antiviral response and plays a pivotal role in combating viruses such as DCV, CrPV, and IIV-6 (17, 19, 25). However, little is known about the sensing and signaling of virus infections that trigger the induced immune responses. In this study, we observed an upregulation of circZfh1 expression in *Drosophila* cells infected with DCV and revealed its antiviral role against DCV infection. Moreover, we identified CRAV as the protein encoded by circZfh1 and demonstrated that its expression enhances the expression of Upd3, thereby activating the JAK-STAT pathway to boost the antiviral response. These results provide insights into the sensing and signaling of the JAK-STAT immune pathway in *Drosophila* cells infected with DCV, a virus known for its potent suppression of the primary antiviral RNAi response.

Although the precise mechanism remains elusive, the Imd pathway has been implicated in antiviral defense in *Drosophila* infected with SINV (20), CrPV (21), and DCV (45, 46), with Zfh1 acting as a negative regulator of the Imd pathway (37). To differentiate between the antiviral effects of Zfh1 and circZfh1, we employed siRNAs that specifically targeted the respective transcripts without inducing significant alterations in the expression of the non-targeted Zfh1 mRNA or circZfh1 (Fig. 1C, E and 2C). Knockdown of Zfh1 in *Drosophila* S2 cells led to increased levels of both DCV and FHV (Fig. 2D and E), along with heightened expression of *AttD* and *DptB* marker genes, indicating activation of the Imd pathway upon Zfh1 knockdown (Fig. S2C). In contrast, we observed no significant alterations in the expression of these marker genes in circZfh1 knockdown cells infected with DCV and FHV (Fig. S2D), implying that circZfh1 restricts virus replication via mechanisms independent of the Imd pathway. Furthermore, we demonstrated that the antiviral function of circZfh1 is mediated by its encoded protein, CRAV, through activating the JAK-STAT pathway.

As viral infections typically hijack the cellular protein machinery to translate viral proteins in a manner that bypasses the need for a cap structure, they can suppress canonical cap-dependent translation (47). This leads to successful virus replication while attenuating the host’s immune response. DCV has a positive single-stranded RNA genome that contains two IRES elements to guide viral protein translation (48). Circular RNAs can facilitate the translation of functional proteins via IRES- or m^6^A-dependent mechanisms (32, 34, 49), indicating that the translation of circZfh1 may also involve similar elements for initiation. Compared with the Zfh1 gene, the reading frame of CRAV undergoes a change, resulting in a unique C-terminal extension of 69 amino acids. Moreover, we noted the presence of CRAV across all species within the melanogaster subgroup, whereas it was absent in species within the closest obscura group. Ectopic expression of these CRAV homologs similarly inhibited DCV replication (Fig. 4D), suggesting conservation of the antiviral function of CRAV. Remarkably, we observed a higher number of mutations in CRAV from *D. simulans* and *D. yakuba*, implying that the generation of circZfh1 was selected for during the virus-host arms race, particularly in the context of viruses such as DCV that have coevolved with their hosts (50).

RNAi is the primary antiviral mechanism in *Drosophila*, effectively suppressing the replication of viruses deficient in VSR, resulting in limited virus replication and preventing the activation of host immune responses (11, 51). We observed that viral RNA levels remain low, with no difference in the replication of FHVΔB2, a VSR B2-deficient FHV, in S2 cells, regardless of the presence of circZfh1 (Fig. 2F and G; Fig. S2E). In contrast, the presence of VSRs such as FHV-B2 or DCV-1A significantly increases FHV RNA1 replication. This increase reveals a marked difference in virus replication between S2 cells with and without circZfh1 knockdown (Fig. 2H), suggesting that circZfh1 or CRAV plays an antiviral role once virus replication becomes effective. Consistently, we observed increased RNA levels of both DCV and FHV, both possessing potent VSRs (DCV-1A and FHV-B2, respectively), in S2 cells following circZfh1 knockdown (Fig. 1D, F and 2A; Fig. S2B). Additionally, the expression levels of circZfh1 and CRAV in cells positively correlate with the concentration of the DCV inoculum (Fig. 1B and 3F; Fig. S3G), suggesting that circZfh1 and CRAV levels depend on virus replication levels, although the detailed mechanisms underlying circZfh1 biogenesis remain unclear. These results suggest that circZfh1 and CRAV play an antiviral role when the virus can replicate efficiently in cells, which is dependent on the presence of VSR to counteract the host RNAi immune response and promote efficient viral replication.

The JAK-STAT pathway was initially characterized in mammals as an important antiviral pathway for its involvement in interferon signal transduction (52–54). During virus infection, JAK-STAT pathway may be induced by tissue damage caused by the virus, as well as by reactive oxygen species (ROS) (19, 25). Both tissue damage and ROS typically induce expression of *upd3*, thereby activating the JAK-STAT pathway (25, 55–57). For example, enterocytes in the *Drosophila* midgut produce cytokines (Upd, Upd2, and Upd3) in response to tissue damage caused by enteric infection, which then activates the JAK-STAT signaling pathway in intestinal stem cells to promote rapid division and replenish the intestinal epithelium (58). The JAK-STAT pathway in *Drosophila* can be activated via the ERK pathway to reduce West Nile virus (WNV) replication through an integrated immune response (59). In *Drosophila* infected with the DNA virus IIV-6, ROS generated by NADPH-oxidase Nox triggers p38 MAPK pathway activation and Upds expression, which subsequently activates JAK-STAT signaling to protect the fly from IIV-6 infection (25). In our study, we demonstrated that the antiviral response mediated by CRAV is associated with the JAK-STAT pathway, rather than the Dcr-2/Vago, STING/Nazo, Toll, or Imd pathways (Fig. 5A). We found that CRAV-induced Upd3 expression activates the JAK-STAT pathway not only in CRAV-expressing cells but also in surrounding cells, thereby conferring resistance to DCV infection (Fig. S5E). However, the detailed mechanism of CRAV-induced activation of the JAK-STAT pathway remains unknown. Further exploration of the antiviral mechanism of CRAV is ongoing, considering all the possibilities mentioned above.

Our findings indicated that knocking down circZfh1 hindered the expression of the JAK-STAT pathway target genes *vir-1* and *TotA* in both cells and fruit flies (Fig. 5D and 6H). However, the exogenous expression of circZfh1 or CRAV in cells stimulated the upregulation of *TotA* but not *vir-1* (Fig. 5E). Similarly, a previous study demonstrated that expression of vir-1 was reduced in *hop* loss-of-function mutant flies but was not triggered in flies expressing the gain-of-function allele *Tum-l* of *hop (19*). These results suggested that the expression level of each JAK-STAT target gene may be determined not only by Stat92E but also by other regulatory factors. Additionally, consistent with previous findings (19), we demonstrated that *vir-1* had no impact on DCV replication; by contrast, downregulating *TotA* in S2 cells significantly increased DCV replication (Fig. S5A and B). Based on these results, it appears that the circZfh1-CRAV-JAK-STAT-TotA signaling cascade confers virus resistance in the presence, but not in the absence, of viral suppression of antiviral RNAi, thereby revealing a new counter-defensive strategy.

## MATERIALS AND METHODS

### Fly strains, husbandry, and infection methods

To generate circZfh1 knockout (KO) flies, the intron between exon 1 and exon 2 of the *zfh1* gene was removed using CRISPR-dependent homologous recombination. The sgRNAs vector skeleton, pCFD3-dU6:3gRNA plasmid, and the donor vector skeleton, pENTY-GAL4 plasmid, were gifts from Prof. Li He, USTC, China. The flanking sequences of the targeted intron were designed as sgRNAs with the following sequences: 5′-GCGCCGTCGTCTTCACGTTT-3′ and 5′-GCTGAATGATGGTATCCTCT-3′ for the upstream site, and 5′-ATTTGTACTGCTCAGTAACT-3′ and 5′-ATTTGTACTGCTCAGTAACT-3′ for the downstream site. To generate the donor plasmid, we PCR amplified a 1,300 bp left homology arm (3R: 30765081…30766380) and a 1,140 bp right homology arm (3R: 30773573…30774712) from genomic DNA of the {nos-Cas9}FFaassttattP40 fly strain (Bloomington #78781). These left and right homology arms were subcloned into pENTY-GAL4 and replaced the GAL4 cassette using the One Step Cloning Kit (Vazyme, Nanjing, China, Cat. No. C115). Four sgRNAs vectors and one donor vector were co-injected into the embryos of {nos-Cas9}attP40 flies. The genome-editing conditions were confirmed by PCR. The offspring of intron homozygotes knockout flies were maintained as the *circZfh1 KO* stock. A sister line without gene editing was used as the wild-type (*WT*) control stock in this study. The WT stock and *circZfh1 KO* stock shared the same genetic background.

Flies were raised on standard cornmeal agar medium at 25°C. All fly stocks were tested for Wolbachia infection and treated with 0.25 mg/mL tetracycline in standard cornmeal agar medium for at least two generations when necessary. All viral infection treatments were performed on 3- to 5-day-old adult flies. For viral survival assays, three groups of 20 male flies from the *WT* and *circZfh1 KO* stocks were injected with 25 nL of DCV (20 TCID50/fly) using a FemtoJET microinjector (Eppendorf, Germany), and survival was monitored on a daily basis. For gene analysis experiments, flies were harvested at the indicated time points, and total RNA was extracted directly using Trizol reagent (Pufei, Shanghai, China, Cat. No. 3101).

### Plasmid construction

The pMT-circZfh1 plasmid shown in Fig. S1J was constructed according to previously described methods (60). The construct was generated in the pMT/V5-His A vector (Invitrogen, USA, Cat. No. V412020). DNA fragments were PCR amplified from the genomic DNA of S2 cells and cloned into pMT/V5-His A using the One Step Cloning Kit (Vazyme, Nanjing, China, Cat. No. C115).

The plasmids depicted in Fig. 3C were generated based on pMT-circZfh1. To prevent translation of the expected protein, the start codon ATG was mutated to TTG at exon 3, resulting in the vector pMT-circZfh1mut. A sequence encoding the V5-tag was inserted either 5' or 3' to the stop codon TGA in exon 3, yielding pMT-V5-TGA and pMT-TGA-V5, respectively. pMT-ATGmut-V5 was constructed by adding a V5-tag encoding sequence 5' to the stop codon TGA of pMT-circZfh1mut.

The CRAV ectopic expression plasmid pMT-CRAV was generated by cloning the linearized full-length ORF of circZfh1. For other protein expression vectors, the coding regions of *Drosophila* proteins were amplified by PCR using gene-specific primers from the cDNA of S2 cells, and PCR fragments were cloned into pMT/V5-His A.

The primers used for plasmid construction are listed in Table S1. All plasmids were confirmed by Sanger sequencing.

### Cell culture and virus infection

*Drosophila* S2 cells were maintained in Schneider’s Insect Medium (Sigma-Aldrich, USA) supplemented with 10% heat-inactivated fetal bovine serum (Gibco, USA), 5 mM sodium bicarbonate (Sigma-Aldrich, USA), 5 mM calcium chloride (Sigma-Aldrich, USA), 100 U/mL penicillin, and 100 µg/mL streptomycin (HyClone, USA) at 25°C.

For virus infection, S2 cells were seeded into 24-well cell culture plates and allowed us to attach for 3 h at 25°C. Diluted virus (DCV [MOI = 1], FHV [MOI = 1]) was then added to each well at a multiplicity of infections. Virus replication or gene expression levels were determined at the indicated times by quantitative reverse transcription polymerase chain reaction (qRT-PCR) using the primers listed in Table S1 for dsRNA synthesis.

### RNA extraction and reverse transcription quantitative PCR

Total RNA was extracted from S2 cells, whole flies, or dissected tissues using TRIzol reagent according to the manufacturer’s instructions. Reverse transcription was performed using the RevertAid First Strand cDNA Synthesis Kit (Thermo Fisher Scientific, USA). Real-time PCR was conducted on a LightCycler 96 System (Roche, Switzerland) with ChamQ Universal SYBR qPCR Master Mix (Vazyme, Nanjing, China) following the manufacturer’s instructions. Primers for qRT-PCR are listed in Table S1. All genes were assayed in triplicate for reproducibility. The expression levels of target genes relative to the control rp49 were calculated using the 2-ΔΔCt method (61).

### Virus titration

The DCV-infected cells were collected at the appropriate time points, and three freeze/thaw cycles were performed before centrifugation at 10,000 × *g* for 5 minutes. The suspensions were titrated by end-point dilution on S2 cells as described (62).

### Plasmid DNA transfection

For the transfection experiment, S2 cells were plated in 12-well plates and grown for several hours to reach 80% confluence. Then, 1 µg of plasmid was transfected into the cells using Lipofectamine 6000 Transfection Reagent (Beyotime, Shanghai, China) according to the manufacturer’s protocol. The cells were stimulated with 25 µM CuSO_4_ at 24 h post-transfection if necessary. Transfected cells were harvested and directly processed to extract total RNA or protein at 2 days post-transfection.

### Identification of differentially expressed circRNAs

To ensure high-quality clean reads for subsequent bioinformatic analysis, we filtered the raw data to remove low-quality reads, adapter contamination, and sequences with a high unknown base content. We also excluded linear RNAs by aligning the clean reads to the *Drosophila* genome (dm6 assembly) using Bowtie2 (63). To identify circular RNA, we extracted the sequences that did not match the genome and used Find_circ (64). We performed quantitative and differential expression analysis of the identified circular RNA using CIRIquant based on gene annotation provided by Flybase (version r6.49) (65). Circular RNAs with log2Foldchange > 1.5 (*P* value < 0.05) and circRNA_bsj value >1 in at least three samples were considered differentially expressed circRNAs. We generated a volcano plot using the R package ggplot2 (version 3.3.5) to visualize the differential expression.

### Gene knockdown by RNAi

For dsRNA-based RNAi, the protocol followed the guidelines provided at https://fgr.hms.harvard.edu. DNA templates for dsRNA synthesis were obtained by PCR using gene-specific primers that contained the T7 polymerase recognition sequence at their 5' end. The primer sequences can be found in Table S1. The dsRNAs targeting each gene were synthesized using a T7 transcription kit (Toyobo, Japan) and visualized by agarose gel electrophoresis. Approximately 1.5 × 10^6^ S2 cells were bathed in 400 µL of serum-free medium containing 7 µg of dsRNA per well of a 12-well plate for 30 minutes at 25°C. The cells were then supplemented with 400 µL of complete medium containing 20% FBS and incubated at 25°C. After 2 days, the cells were harvested to assess knockdown efficiency or used for virus infection.

For siRNA-based RNAi, S2 cells were plated in 12-well plates and allowed to reach 80% confluence. Then, 8 pmol of siRNA was transfected into the cells using Lipofectamine 6000 Transfection Reagent (Beyotime, Shanghai, China) following the manufacturer’s protocol. After culturing at 25°C for 2 days, the cells were harvested to assess knockdown efficiency or used for virus infection. The sequences of siRNAs targeting circRNAs can be found in Table S1.

### RNA-seq library construction and sequencing

For the transcriptomic analysis presented in Fig. S1A, total RNA was extracted from S2 cells infected with DCV (MOI = 1) for 48 h or uninfected cells using TRIzol (Pufei, Shanghai, China). The RNA-seq libraries were constructed and sequenced using BGISEQ-500 at BGI Genomics (Shenzhen, China).

### Establishment of a stable shRNA-expressing cell line

To generate stable S2 cell lines expressing shRNA, we utilized the plasmid pMT-PURO, which contains both the expression cassette and puromycin selection marker (66). We inserted the eGFP encoding sequence downstream of the puromycin coding region, separated by a T2A sequence (5′-GAGGGCAGAGGAAGTCTTCTAACATGCGGTGACGTGGAGGAGAATCCCGGCCCT-3′), resulting in the reporter vector pMT-PURO-T2A-eGFP. Following the approach described by Ni et al. (67), we synthesized a modified microRNA scaffold to generate shRNA targeting the junction site of circZfh1 or a scramble sequence (control) and cloned them into pMT-PURO-T2A-eGFP. The resulting shRNA expression vector or the control vector was transfected into S2 cells using Lipofectamine 6000 Transfection Reagent (Beyotime, Shanghai, China) according to the manufacturer’s protocol. Stable transformants were screened as previously described (66), and the enrichment efficiency was determined by observing eGFP expression under a fluorescence microscope (Olympus IX73, Japan).

### Western blot analysis

S2 cells from a single well of a 12-well plate were harvested and washed twice with ice-cold PBS buffer. Cell lysis was performed by adding 80 µL of cold lysis buffer (20 mM Tris-HCl [pH 7.0], 50 mM NaCl, 0.5 mM EDTA, 0.5% NP40, 0.5% sodium deoxycholate, 1 mM PMSF) and keeping the samples on ice for 60 minutes. The cell extracts were then centrifuged at 12,000 rpm for 15 minutes at 4°C, and the supernatants were collected as total protein extracts. The protein extracts were separated by SDS-PAGE and transferred to a PVDF membrane (Millipore, USA). The membrane was blocked and then incubated with the designated primary antibodies overnight at 4°C. After washing, the membranes were incubated with HRP-labeled goat anti-mouse/rabbit IgG secondary antibody (Beyotime, Shanghai, China) for 1 h. Immunoreactivity was detected using ECL substrates (Beyotime, Shanghai, China) and analyzed with LAS4000 (GE Healthcare, USA). Quantification of the western blot bands was performed using ImageJ software (http://rsb.info.nih.gov/ij/index.html).

In this study, the following commercial antibodies were used: anti-β-tubulin (1:5,000, CWbio, Jiangsu, China), anti-V5 (1:10,000, Invitrogen, USA).

### Polyclonal antibodies against CRAV

Polyclonal antibodies against CRAV were generated by GenScript Biotech Corporation (Nanjing, China) to immunize rabbits with two chemically synthesized peptides corresponding to amino acids 4–17 and 217–230 of CRAV, resulting in the production of Ab4 and Ab217 antibodies, respectively. Additionally, the coding region of CRAV was cloned into a pET-MBP vector and transformed into *E. coli* BL21. Recombinant CRAV protein expression was induced in *E. coli* with 0.2 mM IPTG at 16°C for 4 h, followed by purification of soluble recombinant CRAV protein using a HiTrap IMAC column (GE Healthcare, USA). The Ab1 antibody, a mouse polyclonal antibody against CRAV, was generated by immunizing mice with the purified recombinant CRAV protein using a published protocol (68).

### RNA fluorescence *in situ* hybridization (FISH)

For RNA FISH, *in vitro*-transcribed antisense probes labeled with Alexa Fluor 488/546 (Thermo Fisher Scientific, USA) were used. The RNA FISH procedure was carried out as previously described (69). Briefly, S2 cells were inoculated on slides and cultured in 24-well plates. The slides were pre-treated with 0.5 mg/mL ConA (Sangon Biotech, Shanghai, China) for 3 h at room temperature, followed by drying. After allowing the cells to attach at 25°C for 3 h, they were washed with PBS and fixed with 37% formaldehyde at room temperature for 10 minutes. Fixed cells were then dehydrated in 70%, 90%, and 100% ethanol for 5 minutes each and air-dried. Denatured probes (5 µL in 40 µL hybridization buffer containing 75% formamide, 2× SSC, 15% dextran sulfate) were applied onto the coverslips and hybridized overnight at 37°C. After *in situ* hybridization, slides were washed twice for 5 minutes each with 2× SSC containing 50% formamide at 45°C, followed by two washes with 2× SSC at 45°C. Nuclei were counterstained with 500 ng/mL DAPI (Biosharp, Hefei, China), and fluorescence images were captured using a confocal fluorescence microscope (Zeiss LSM 880, Germany).

### Immunofluorescence

For flies, internal tissues were dissected and fixed in 4% paraformaldehyde in PBS for 15 minutes, followed by washing in PBS. Then, the samples were incubated with 3% BSA in 1× PBST (0.1% Triton X-100 in PBS) for 1 h at room temperature. Samples were then incubated overnight with primary antibody (Ab1, 1:50) at 4°C. After washing with 1× PBST three times, the samples were incubated with goat anti-mouse IgG H&L (Alexa Fluor 488, Abcam, USA) for 1 h at room temperature. The nuclei were stained with 500 ng/mL DAPI (Biosharp, Hefei, China). Samples were washed in 1× PBST, dissected, and placed on slides. A confocal fluorescence microscope (Zeiss LSM 880, Germany) was used to capture the images.

### Immunoprecipitation

S2 cells were transfected with the relevant plasmids and cultured for 48 h. Transfected cells were collected and washed with PBS. The cells were then lysed in cell lysis buffer (50 mM Tris HCl [pH 7.4], 150 mM NaCl, 1 mM EDTA, 1% Triton X-100, and 1 mM PMSF) on ice for 1 h. After centrifugation at 12,000 rpm for 15 minutes at 4°C, the lysates were incubated overnight at 4°C with either Anti-Flag M2 Affinity Gel (Sigma-Aldrich, USA) or Protein A/G Agarose beads (Santa Cruz, USA) conjugated with anti-V5 antibody (Invitrogen, USA). The immunoprecipitates were then washed five times with cell lysis buffer and subjected to western blot analysis.

### Statistical analysis

Each experiment was repeated independently at least three times with similar results. The western blotting images are representative of three independent experiments. Unless stated otherwise, the results of quantitative experiments are reported as the mean ± SD of three independent experiments. Statistical analysis was performed using an unpaired two-tailed Student’s *t*-test for comparisons between two groups. Survival curves were plotted and analyzed using the log-rank test (Kaplan-Meier method) with GraphPad Prism software. Statistical significance was defined as follows: n.s., not significant; *P < 0.05; **P < 0.01; and ***P < 0.001. Error bars represent standard deviation (SD) from triplicate experiments.

## SUPPLEMENTAL MATERIAL

Supplemental Figures mbio.01469-24-s0001.pdf Fig. S1-S5.

Table S1 mbio.01469-24-s0002.pdf Primers and sequences.

ASM does not own the copyrights to Supplemental Material that may be linked to, or accessed through, an article. The authors have granted ASM a non-exclusive, world-wide license to publish the Supplemental Material files. Please contact the corresponding author directly for reuse.
