# Supplementary material for: Protein-coding circular RNA enhances antiviral immunity via JAK/STAT pathway in Drosophila
Source: mBio. 2024 Aug 19;15(9):e01469-24. doi: 10.1128/mbio.01469-24 (PMC11389369; doi:10.1128/mbio.01469-24)
Supplement: Supplemental Figures — Fig. S1-S5. [file mbio.01469-24-s0001.pdf]

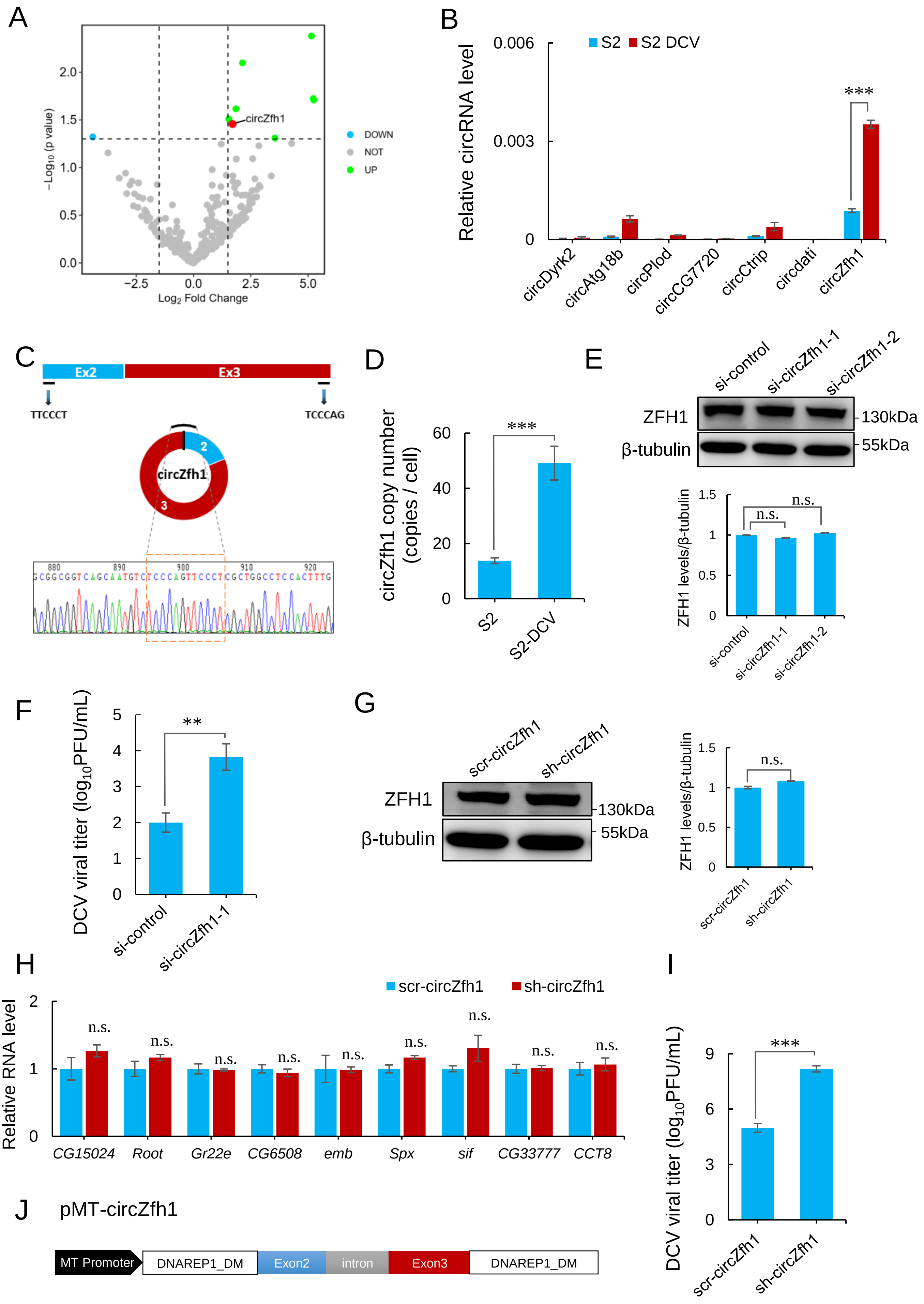

A
2.0
1.5
1.0
0.5
0.0
−Log10 (p value)
-2.5
0.0
2.5
5.0
Log2 Fold Change
circZfh1
DOWN
NOT
UP
B
Relative circRNA level
0.006
0.003
0
S2
S2 DCV
***
circDyrk2
circAtg18b
circPlod
circCG7720
circCtrip
circdati
circZfh1
C
Ex2
Ex3
TTCCCT
TCCCAG
circZfh1
2
3
880
890
900
910
920
D
circZfh1 copy number (copies / cell)
60
40
20
0
***
S2
S2-DCV
E
si-control
si-circZfh1-1
si-circZfh1-2
ZFH1
β-tubulin
130kDa
55kDa
ZFH1 levels/β-tubulin
1.5
1
0.5
0
n.s.
n.s.
F
DCV viral titer (log10PFU/mL)
5
4
3
2
1
0
**
si-control
si-circZfh1-1
G
scr-circZfh1
sh-circZfh1
ZFH1
β-tubulin
130kDa
55kDa
ZFH1 levels/β-tubulin
1.5
1
0.5
0
n.s.
H
Relative RNA level
2
1
0
scr-circZfh1
sh-circZfh1
n.s.
CG15024
Root
Gr22e
CG6508
emb
Spx
sif
CG33777
CCT8
I
DCV viral titer (log10PFU/mL)
9
6
3
0
***
scr-circZfh1
sh-circZfh1
J
pMT-circZfh1
MT Promoter
DNAREP1_DM
Exon2
intron
Exon3
DNAREP1_DM

Fig. S1. Detection and validation of circZfh1 in *Drosophila* S2 cells, related to Fig. 1.

(**A**) Volcano map illustrating the differential expression of circRNAs in *Drosophila* S2 cells with or without DCV infection, using |log2FC|=1.5 and p value=0.05 as the cut-off value. Upregulated circRNAs are shown as green dots, and downregulated circRNAs are shown as blue dots. CircZfh1 is highlighted in red. (**B**) RT-qPCR analysis of seven upregulated circRNAs in DCV-infected S2 cells compared to uninfected S2 cells, except for another one of the circRNAs predicted could not be detected. (**C**) PCR amplification and Sanger sequencing of the circZfh1 junction sequence. (**D**) The copy number of circZfh1 in S2 cells with or without DCV (MOI=1) infection was determined by RT-qPCR at 48 hpi. . (**E**) Western blot for ZFH1 in S2 cells transfected with si-control, si-circZfh1-1 or si-circZfh1-2 (top). The bar graph indicates the relative fold change calculated from the average protein levels, normalized to β-tubulin (bottom). (**F**) Determination of viral titers in si-control or si-circZfh1-1 cells infected with DCV (MOI=1) at 48 hpi. (**G**) Western blot for ZFH1 in scr-circZfh1 or sh-circZfh1 cells (left). The bar graph indicates the relative fold change calculated from the average protein levels, normalized to β-tubulin (right). (**H**) RT-qPCR analysis of predicted off-target genes expression in sh-circZfh1 and scr-circZfh1 cells. (**I**) Determination of viral titers in scr-circZfh1 or sh-circZfh1 cells infected with DCV (MOI=1) at 48 hpi. (**J**) Schematic representation of the circZfh1 exogenous expression plasmid constructed. Mean ± SD of three independent experiments is shown (**B, D-I**); Statistical analysis was performed for (**B, D-I**), ** $p < 0.01$; *** $p < 0.001$; n.s., not significant (Student's *t*-test). The representatives of triplicate experiments were shown (**E, G**).

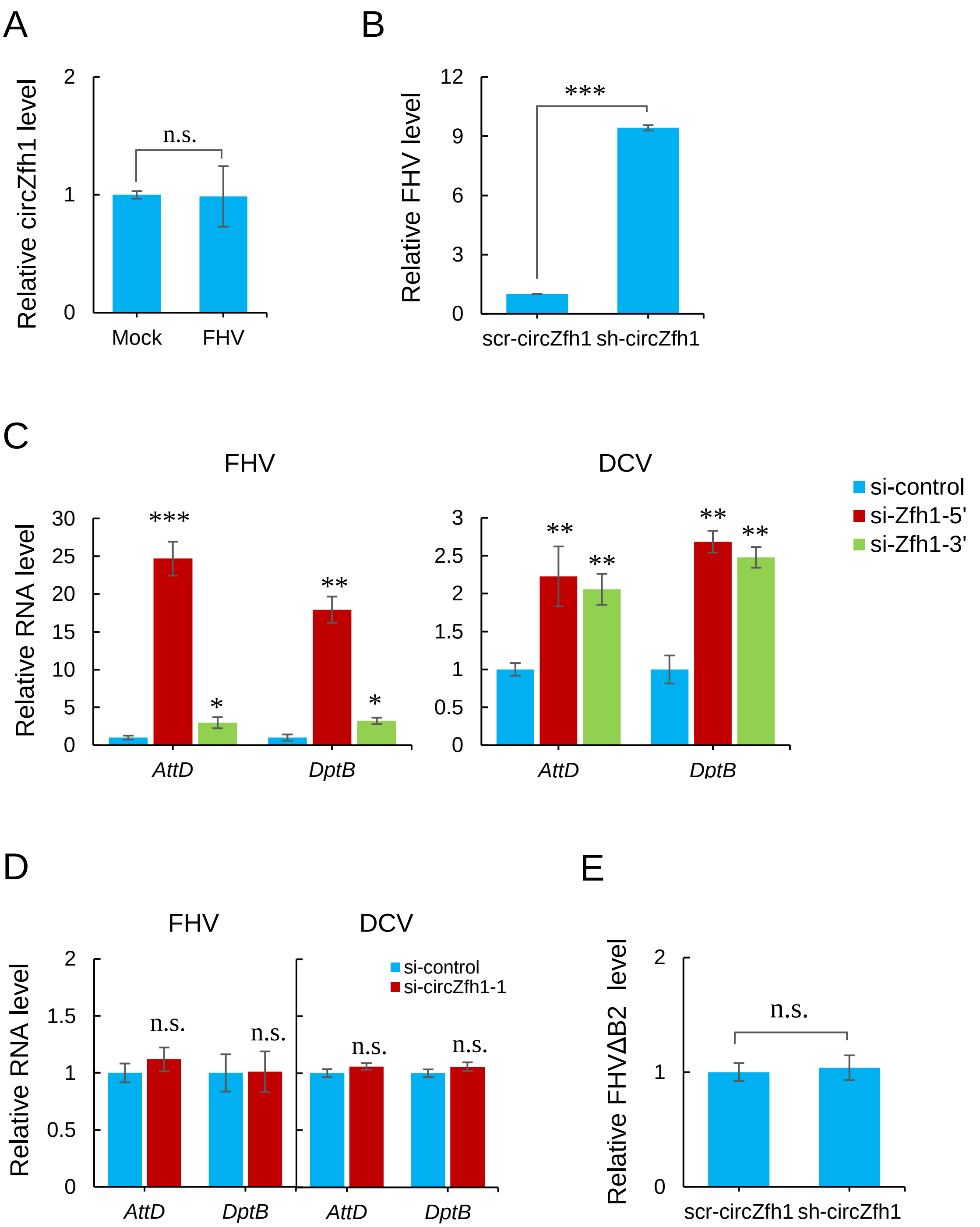

Fig. S2. The antiviral effect of circZfh1 in *Drosophila* S2 cells, related to Fig. 2.

(**A**) RT-qPCR analysis of circZfh1 expression levels, normalized to *rp49*, in S2 cells infected with FHV at 48 hpi. (**B**) RT-qPCR analysis of relative FHV RNA levels, normalized to *rp49,* in scr-circZfh1 or sh-circZfh1 cells infected with FHV at 48 hpi. (**C**) RT-qPCR analysis of relative mRNA levels of *AttD*, *DptB* in S2 cells transfected with control siRNA or two siRNAs targeting Zfh1 and infected with FHV or DCV for 48 hrs. (**D**) RT-qPCR analysis of AMP genes (*AttD*, *DptB*) expression levels, normalized to *rp49*, in S2 cells transfected with si-control or si-circZfh1-1 and infected with FHV or DCV for 48 hours. (**E**) RT-qPCR analysis of relative FHVΔB2 levels, normalized to *rp49,* in scr-circZfh1 or sh-circZfh1 cells infected with FHVΔB2 at 24 hpi. Mean ± SD of three independent experiments is shown (**A-E**); Statistical analysis was performed for (**A -E**); * $p < 0.05$;** $p < 0.01$; *** $p < 0.001$; n.s., not significant (Student's *t*-test).

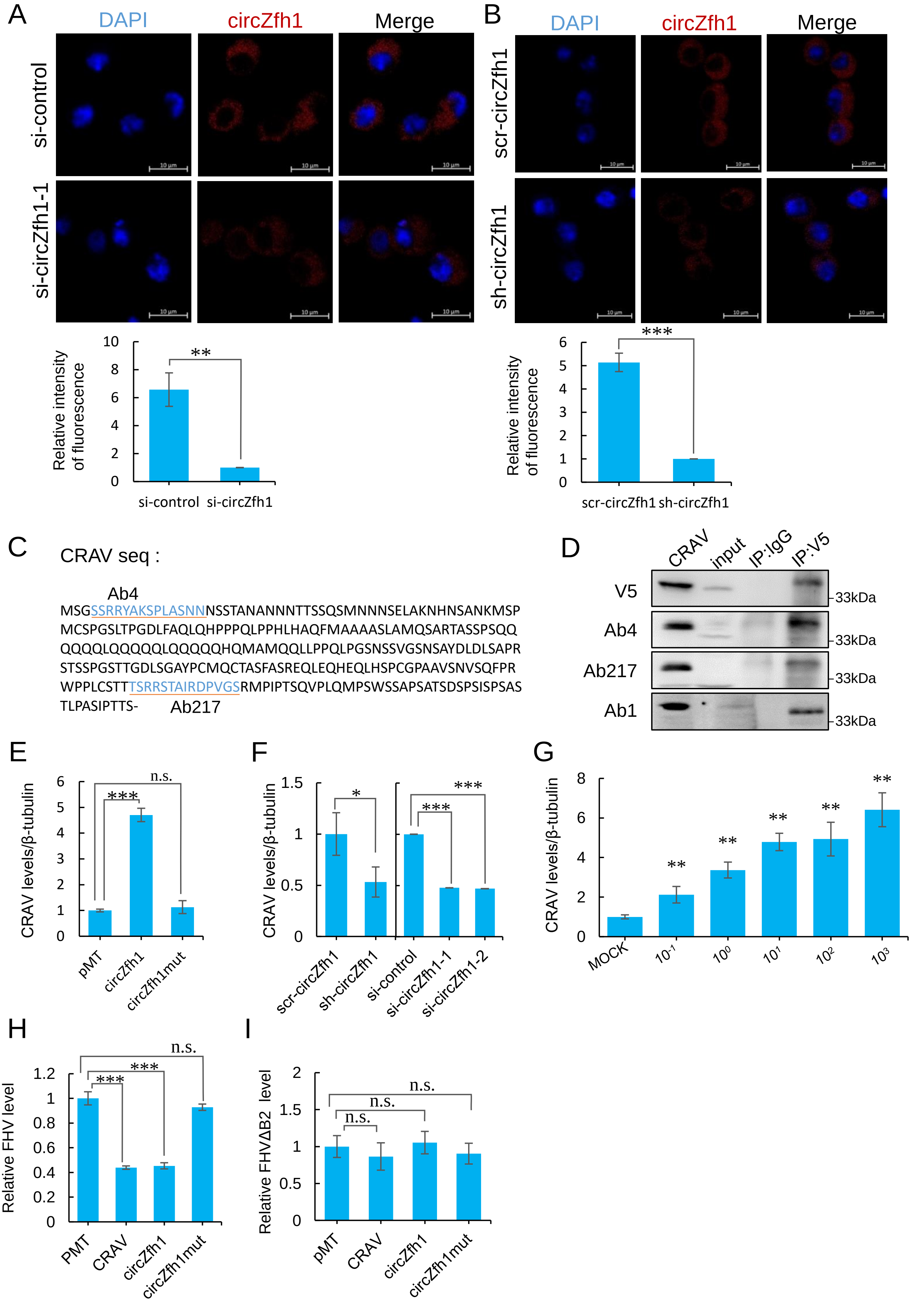
A
DAPI
circZfh1
Merge
si-control
si-circZfh1-1
10 μm
Relative intensity of fluorescence
**
si-control
si-circZfh1
B
DAPI
circZfh1
Merge
scr-circZfh1
sh-circZfh1
10 μm
Relative intensity of fluorescence
***
scr-circZfh1
sh-circZfh1
C
CRAV seq :
Ab4
MSGSSRRYAKSPLASNNNSSTANANNNTTSSQSMNNNSELAKNHNSANKMSP
MCSPGSLTPGDLFAQLQHPPPQLPPHLHAQFMAAAASLAMQSARTASSPSQQ
QQQQLQQQQQLQQQQQHQMAMQQLLPPQLPGSNSSVGSNSAYDLDLSAPR
STSSPGSTTGDLSGAYPCMQCTASFASREQLEQHEQLHSPCGPAAVSNVSQFPR
WPPLCSTTTSRRSTAIRDPVGSRMPIPTSQVPLQMPSWSSAPSATSDSPSISPSAS
TLPASIPTTS-
Ab217
D
CRAV
input
IP:IgG
IP:V5
V5
Ab4
Ab217
Ab1
33kDa
E
CRAV levels/β-tubulin
***
n.s.
pMT
circZfh1
circZfh1mut
F
CRAV levels/β-tubulin
*
***
***
scr-circZfh1
sh-circZfh1
si-control
si-circZfh1-1
si-circZfh1-2
G
CRAV levels/β-tubulin
**
MOCK
10⁻¹
10⁰
10¹
10²
10³
H
Relative FHV level
***
***
n.s.
PMT
CRAV
circZfh1
circZfh1mut
I
Relative FHVΔB2 level
n.s.
n.s.
n.s.
pMT
CRAV
circZfh1
circZfh1mut

Fig. S3. Characterization of CRAV protein encoded by circZfh1, related to Fig. 3.

(**A, B**) FISH results showing the subcellular localization of circZfh1 in S2 cells transfected with si-circZfh1-1 and si-control (top), the bar graph indicates the relative intensity of fluorescence for the FISH results in siRNA treated cells (bottom) (**A**), sh-circZfh1 and scr-circZfh1 cells (top), the bar graph indicates the relative intensity of fluorescence for the FISH results in shRNA cells (bottom) (**B**). Red fluorescence signal indicates circZfh1, and blue fluorescence signal indicates the nuclear location stained by DAPI. Scale bar = 10μm. (**C**) Amino acid sequences of CRAV and the immunogenic peptides used to produce Ab4 and Ab217 antibodies. (**D**) S2 cells transfected with V5-TGA subjected to IP using anti-V5 antibody and Western blot for the IP pellets using anti-V5, Ab4, Ab217, and Ab1 antibodies, respectively. (**E**) The bar graph indicates the relative fold change calculated from the average protein levels for CRAV protein quantification in Fig. 3D, normalized to β-tubulin. (**F**) The bar graph indicates the relative fold change calculated from the average protein levels for CRAV protein quantification in Fig. 3E, normalized to β-tubulin. (**G**) The bar graph indicates the relative fold change calculated from the average protein levels for CRAV protein quantification in Fig. 3F, normalized to β-tubulin. (**H**) S2 cells transfected with pMT, pMT-CRAV, pMT-circZfh1 or pMT-circZfh1mut plasmids were infected with FHV, followed by RT-qPCR analysis of FHV RNA levels, normalized to *rp49*, at 48 hpi. (**I**) S2 cells transfected with pMT, pMT-CRAV, pMT-circZfh1 or pMT-circZfh1mut plasmids were infected with FHVΔB2, followed by RT-qPCR analysis of FHVΔB2 RNA levels, normalized to *rp49*, at 24 hpi. Mean ± SD of three independent experiments is shown (**A-B**, **E-I**); Statistical analysis was performed for (**A-B**, **E-I**); * $p < 0.05$; ** $p < 0.01$; *** $p < 0.001$; n.s., not significant (Student's *t*-test). The representatives of triplicate experiments were shown (**D**).

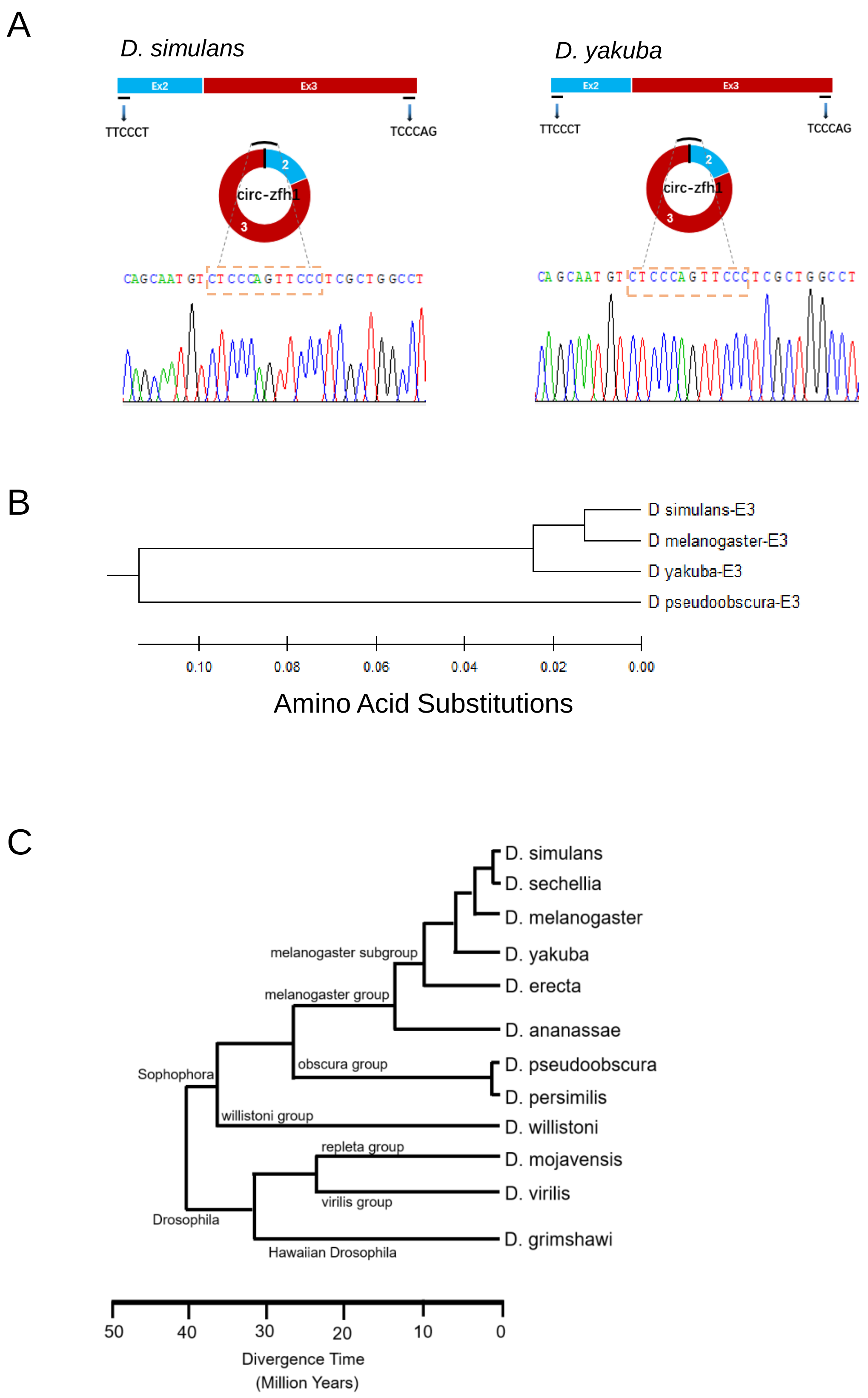

Fig. S4. Characterization of circZfh1 in species within the *melanogaster* subgroup, related to Fig. 4.

(**A**) PCR amplification and Sanger sequencing of the circZfh1 junction sequence in *D. simulans* and *D. yakuba*. (**B**) Phylogenetic analysis of exon3-encoded protein fragments of ZFH1 in *D. simulans*, *D. melanogaster*, *D. yakuba* and *D. pseudoobscura*. (**C**) The phylogenetic tree of 12 species within the family *Drosophilidae*.

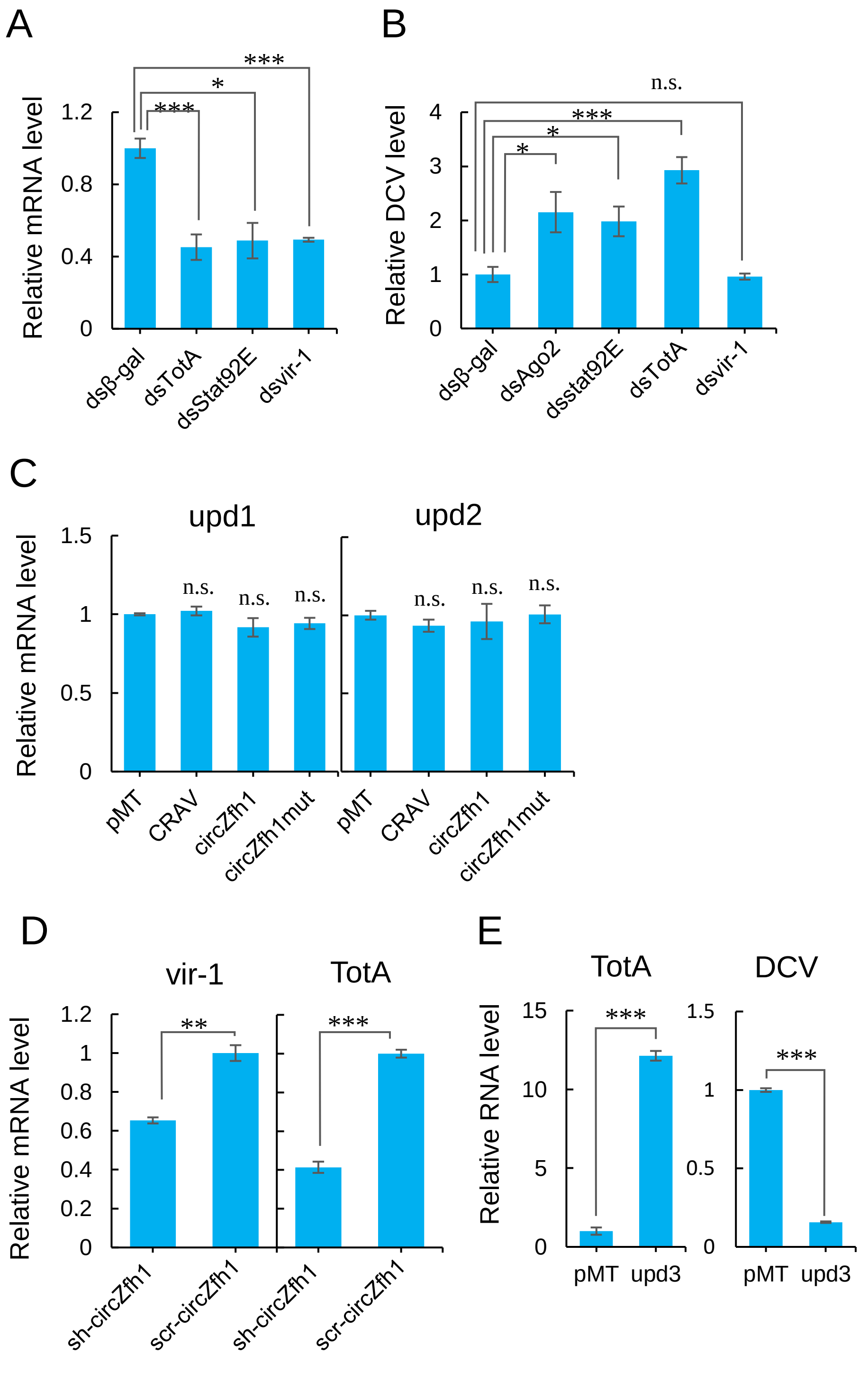

Fig.S5. Activation of the JAK-STAT pathway by CRAV boosts the immune response, related to Fig. 5. (**A**) RT-qPCR analysis of the indicated gene mRNA levels relative to the control in S2 cells pretreated with dsRNAs against a control (*β-gal*), *TotA*, *Stat92E*, or *vir-1*. (**B**) RT-qPCR analysis of relative DCV RNA levels at 48 hpi, normalized to *rp49*, in S2 cells pretreated with dsRNAs against a control (*β-gal*), *TotA*, *Stat92E*, or *vir-1* and infected with DCV. (**C**) RT-qPCR analysis of *upd1* and *upd2* mRNA levels in S2 cells transfected with pMT, pMT-CRAV, pMT-circZfh1, or pMT-circZfh1mut. (**D**) RT-qPCR analysis of *vir-1* and *TotA* mRNA levels in sh-circZfh1 or scr-circZfh1 cells. (**E**) S2 cells transfected with pMT or pMT-upd3 were cultured for 48 h. Then, the supernatant of the cell culture medium was collected and added to the culture medium of naive S2 cells for 24 h, and the S2 cells were infected with DCV prior to RT-qPCR to determine the relative mRNA levels of *TotA* (left) and the DCV level at 48 hpi (right). Mean ± SD of three independent experiments is shown (**A-E**); Statistical analysis was performed for (**A-E**); * $p < 0.05$; ** $p < 0.01$; *** $p < 0.001$; n.s., not significant (Student's *t*-test).
