# Supplementary material for: Protein-coding circular RNA enhances antiviral immunity via JAK/STAT pathway in Drosophila
Source: mBio. 2024 Aug 19;15(9):e01469-24. doi: 10.1128/mbio.01469-24 (PMC11389369; doi:10.1128/mbio.01469-24)
Supplement: Table S1 — Primers and sequences. [file mbio.01469-24-s0002.pdf]

Table S1: Primers and sequences used in this study

**Sequences of primers used in FISH**	
**primer name**	**sequence(5'-3')**
FISH-circZfh1-probe-Fw	CGGACCGGCGGCGGTCAGCAATGTCTCCCAGTTCCCTCGCTGGCCTCCACTT TGCTCCACCACACCCTATAGTGAGTCGTATTA
FISH-circZfh1-probe-Rv	TAATACGACTCACTATAGGGTGTGGTGGAGCAAAGTGGAGGCCAGCGAGGGA ACTGGGAGACATTGCTGACCGCCGCCGGTCCG
FISH-Zfh1 mRNA-Fw(exon4/5)	ACTCCTCGCAGTGGCCAGGCCTTT
FISH-Zfh1 mRNA-Rv(exon4/5)	TAATACGACTCACTATAGGGAGGCACTTGGAGCACTGAAAGGGC
**Sequences of primers used in plasmid construction**	
**primer name**	**sequence(5'-3')**
pMT-circZfh1-Fw	tattttttttattttatGCAGAAAGGTACCTTCCCTCGCTGGCCTCCACT
pMT-circZfh1--Rv	CTGGGAGACATTGCTGACCGCC
pMT-CRAV-Fw	TCTAGATCGGGGTACCATGAGCGGCAGCAGCAGGCGGT
pMT-CRAV-Rv	GATATCTGCAGAATTCTCAGCTTGTCGTGGGGATGCTC
pMT-circZfh1mut-Fw	ACTTGAGCGGCAGCAGCAGGCGGT
pMT-circZfh1mut-Rv	TGCTGCCGCTCAAGTTGCTCTGCT
pMT-V5-TGA-Fw	TAACCCTCTCCTCGGTCTCGATTCTACGTGAACTGCGGAGCTGCCCA G
pMT-V5-TGA-Rv	CCGAGGAGAGGGTTAGGGATAGGCTTACCGCTTGTCGTGGGGATGCT CG
pMT-TGA-V5-Fw	TAACCCTCTCCTCGGTCTCGATTCTACGACTGCGGAGCTGCCCAGCC G
pMT-TGA-V5-Rv	CCGAGGAGAGGGTTAGGGATAGGCTTACCTCAGCTTGTCGTGGGGATGCTC
**Sequences of primers used in circZfh1 identification**	
**primer name**	**sequence(5'-3')**
divergent primer Fw	CTGGCCATGCAGTCCGCCCGC
divergent primer Rv	TCCTGGATCCCACTGGATCC
convergent primer Fw	ACAAGCGATTCCCCGAGTAT
convergent primer Rv	TGTGGTTCTTGGCCAGCTCG
exon1 primer Fw	GTCTTCTCGCTTCGGTCAAGAAGACACCATC

intron1 primer Fw	TCTCTCTGTGGATTCTGCAGC
intron2 primer Rv	ACTGCTCACTTACTAGCCTTTTGTTGG
**Sequences of primers used in circZfh1 knockdown**	
circZfh1 siRNA-1 Fw	CAAUGUCUCCCAGUUCCCUTT
circZfh1 siRNA-1 Rv	AGGGAACUGGGAGACAUUGTT
circZfh1 siRNA-2 Fw	AUGUCUCCCAGUUCCCUCGTT
circZfh1 siRNA-2 Rv	CGAGGGAACUGGGAGACAUTT
circZfh1 si-control Fw	ACGCUUCUACCGUACUCCUTT
circZfh1 si-control Rv	AGGAGUACGGUAGAAGCGUTT
circZfh1-shRNA-F	cagtCAATGTCTCCCAGTTCCCTCGtagttatattcaagcataCGAGGGAACTGG GAGACATTGgcg
circZfh1-shRNA-R	aattcgcCAATGTCTCCCAGTTCCCTCGtatgcttgaatataactaCGAGGGAACT GGGAGACATTGactGGTAC
circZfh1- scramble-F	cagtCGACGCTTCTACCGTACTCCTtagttatattcaagcataAGGAGTACGGTA GAAGCGTCGgcg
circZfh1- scramble-R	aattcgcCGACGCTTCTACCGTACTCCTtatgcttgaatataactaAGGAGTACGG TAGAAGCGTCGactGGTAC
**Sequences of primers used in RNAi knockdown**	
**primer name**	**sequence(5'-3')**
dsTotA-Fw	TAATACGACTCACTATAGGGATTCCGACGAAGATCGTGAG
dsTotA-Rv	TAATACGACTCACTATAGGGGCAAGCTTTGAACCCAATTC
dsStat92E-Fw	TAATACGACTCACTATAGGGAAGCTGCTTGCCCAAAACTA
dsStat92E-Rv	TAATACGACTCACTATAGGGGTCGACGATAAAGGCAGAGC
dsvir-1-Fw	TAATACGACTCACTATAGGGGAAGCTCTTGTTCGGTTTGC
dsvir-1-Rv	TAATACGACTCACTATAGGGCGCTGCCATCACTAGAATCA
**Sequences of primers used in qPCR**	
**primer name**	**sequence(5'-3')**
RP49-qFw	AGCATACAGGCCCAAGATCG
RP49-qRv	TGTTGTCGATACCCTTGGGC
circDyrk2-qFw	CTGAGTCCGGTGCTTAACTGGT

circDyrk2-qRv	GAATATCCTCGATGAGCTGCGC
circAtg18b-qFw	GAACAGGCGCTCCACGATTCGG
circAtg18b-qRv	TTGCCTCACTGGTCTTCTCGGC
circPlod-qFw	GGAGCAATAGCTTCGCGCAGAA
circPlod-qRv	GATATCCTTGCAGGCAATTCGT
circCG7720-qFw	CGGTGAGGGCTATAAACAGTAC
circCG7720-qRv	TACTACCGCGGATCGGCCATGT
circCtrip-qFw	CGGACGACGTTGTTGCTGTTGT
circCtrip-qRv	TCTCTACCTCCCGATCTCTGCC
circDati-qFw	AGATTTGCCAGCTCGCTGGGCT
circDati-qRv	TTCGCAGGGTCAGCAGTCCCCT
circmgl-qFw	GAGAGACGGAGATGGCATCTTG
circmgl-qRv	TCGTCAGCGCGGCCAGTTGACA
circZfh1-qFw	CGGTCAGCAATGTCTCCCA
circZfh1-qRv	GCTGGTTGGGATGGGCAT
Zfh1 mRNA-qFw	GGTCAGCAATGTCTCCCAGA
Zfh1 mRNA-qRv	TGGTGCTTGAACTTGAAGGC
DCV-qFw	TTTAGCAGATGATGCGGCA
DCV-qRv	GGAGACCAATTTGAGTCGATC
FHV-qFw	TGACCGACAAGAATGAACT
FHV-qRv	TGTCCCATACCGATAAGC
vir-1-qFw	GATCCCAATTTTCCCATCAA
vir-1-qRv	GATTACAGCTGGGTGCACAA
Socs36E-qFw	GCCAACTAGCCAAAAGTAACG
Socs36E-qRv	TGCTGAGAACTTGCTAAGGTG
upd3-qFw	AGCCGGAGCGGTAACAAAA

upd3-qFv	CGAGTAAGATCAGTGACCAGTTC
TotA-qFw	CCCAGTTTGACCCCTGAG
TotA-qRv	GCCCTTCACACCTGGAGA
**Sequences of primers used in circZfh1 KO fly construction**	
**primer name**	**sequence(5'-3')**
left homology arm-Fw	AAAAAGCAGGCTTCGGTACCGCGATATGCATACATATGTG
left homology arm-Rv	ACTGCATGGCGAAGGGGGAG
right homology arm-Fw	CTCCCCCTTCGCCATGCAGTTTCCCTCGCTGGCCTCCAC
right homology arm-Rv	AAGAAAGCTGGGTGTCTAGATTGTGTCCAACTCACCTGGG
